# Supplementary material for: Enrichment of low-frequency functional variants revealed by whole-genome sequencing of multiple isolated European populations
Source: Nat Commun. 2017 Jun 23;8:15927. doi: 10.1038/ncomms15927 (PMC5490002; doi:10.1038/ncomms15927)
Supplement: Supplementary Information [file ncomms15927-s1.pdf]

Type of file: PDF
Size of file: 0 KB
Title of file for HTML: Supplementary Information
Description: Supplementary Figures, Supplementary Tables, Supplementary Notes and Supplementary References

Type of file: pdf
File size:
Title of file for HTML: Peer Review File
Description:

Supplementary Figures

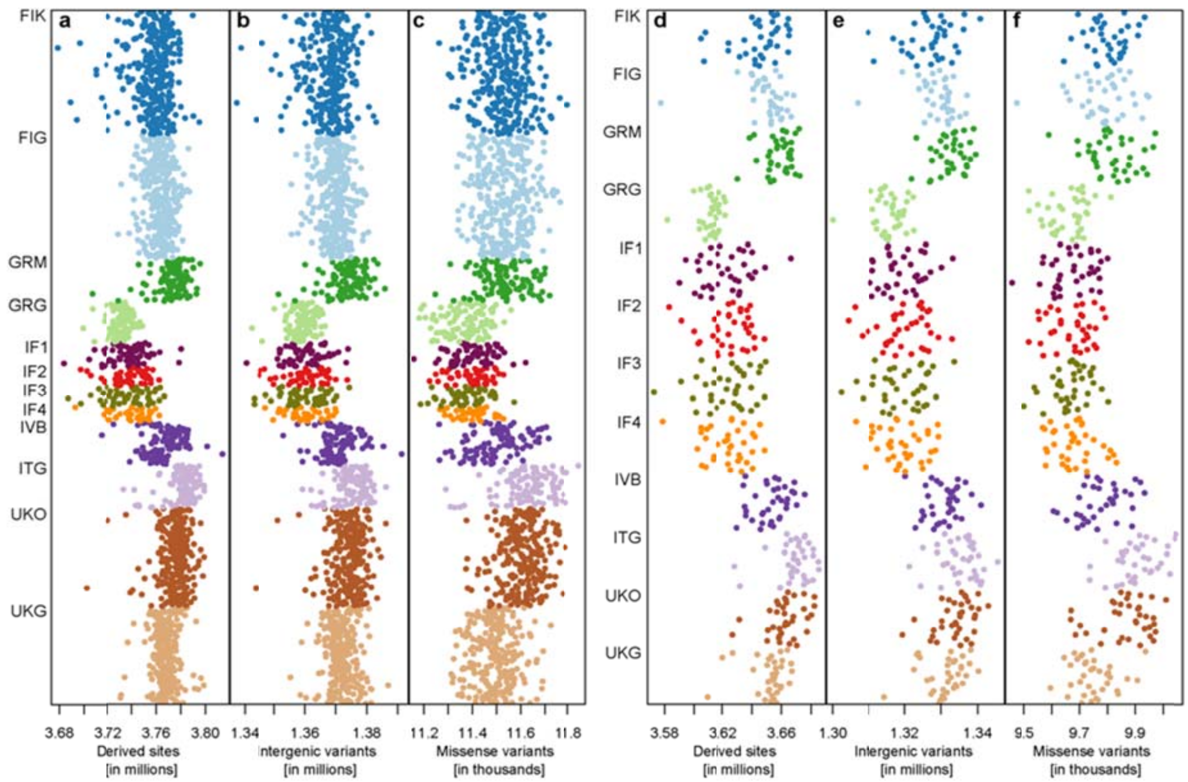

Supplementary Figure 1. Variant sites based on derived allele count in each individual. Left panels (a, b, c) are based on the matched-sample-size and right panels (d, e, f) are based on the minimum-sample-size. The x-axis shows the number of variant sites, and each individual is plotted on the y-axis. The numbers are slightly higher for most of the categories using the sample-size-matched dataset compared with the minimum-sample-size dataset, as the rare variant numbers increase when samples size increase, but the alternative alleles of some these variants are ancestral, which will increase the derived site counts in most of the individuals. We found that singleton counts decrease as sample sizes increase, which is as expected. More variation of the counts is seen in the general populations than in the isolates. Overall, only the Friuli Venezia Giulia isolates - IF1, IF2, IF3 and IF4 - showed lower total variant counts compared to the general population, as is expected. All other isolates showed the opposite pattern, which is most likely due to the ascertainment in sample selection by maximizing the haplotype diversity in each population. GRG showed very low variant counts, which is due to the different variant calling procedure in this population.

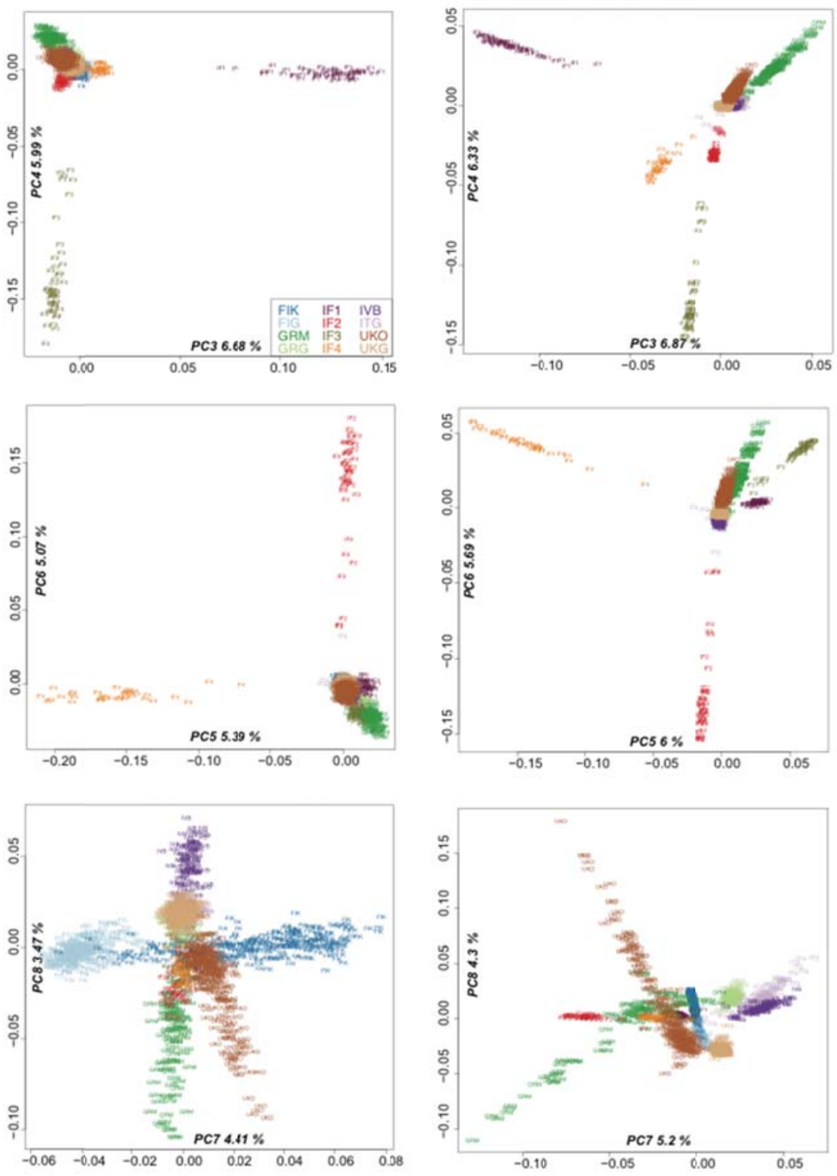

Supplementary Figure 2. PCA plots based on pruned variant dataset with r^2 <0.4 comparing common variants (MAF >0.05 across all the populations; left) with rare variants (MAF across all the populations ranging between 0.01 and 0.05; right). We used the whole dataset for these analyses, and PCAs were performed using EIGENSTRAT v.501[1]. Populations mostly clustered on the PCA according to their geographic locations, and the isolates were positioned close to their corresponding general populations: for example, UKO next to UKG; FIK next to FIG, while all populations from Italy clustered together. The most interesting and striking finding is that the PCA with rare variants separated the populations with higher resolution than that with common variants, especially for the isolated populations from Italy. For example, PC3 and PC4 with rare variants show well-differentiated clusters for IF1, IF2, IF3 and IF4. PC7 and PC8 show additional differentiation for FIK, GRM, IVB and UKO (right panel).

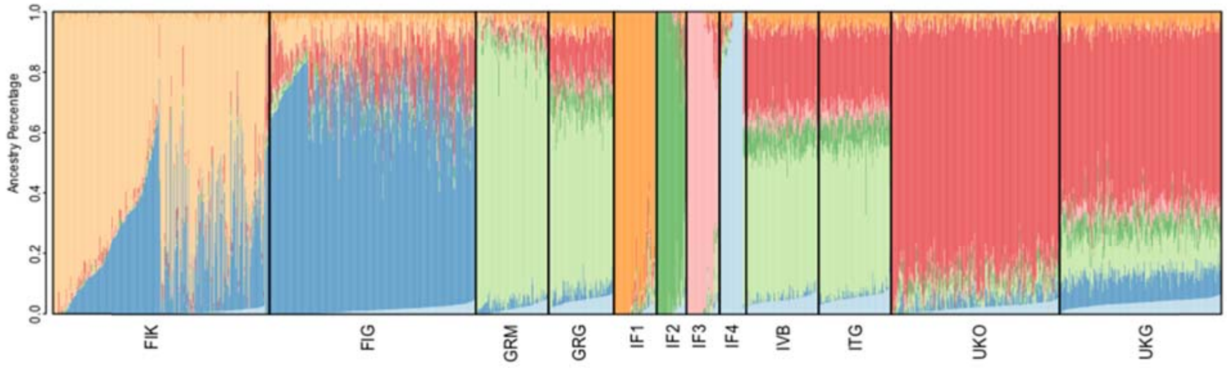

Supplementary Figure 3. ADMIXTURE graph of the populations studied here with K=8. Shared ancestry between the populations studied here was evaluated using ADMIXTURE v1.22[2], with the whole dataset. The optimal number of clusters was assessed through the cross-validation error procedure[2]. Each ADMIXTURE run was replicated five times with different random seeds. Each isolate shared ancestry components with its closest general population.

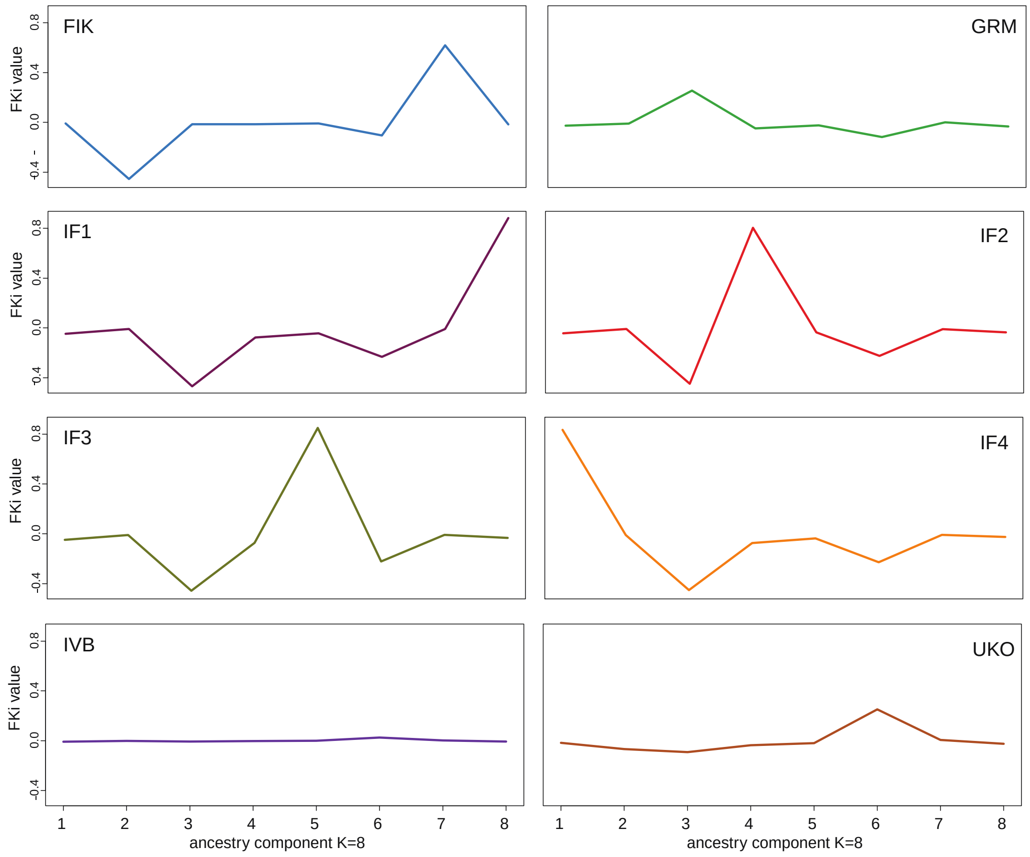

Supplementary Figure 4. FKi values for each population for the ADMIXTURE run with K=8. FKi= $fki_{-isolate}$-$fki_{-general}$ while fki (i = 1, 2, …K) is the mean percentage of each ancestry component in each population. At least one ancestry component differed substantially in frequency compared with the general population. The isolates IF1, IF2, IF3 and IF4 have the largest difference compared with their general population ITG, while IVB has the least difference. This could be due to a more pronounced bottleneck in the history of the four IF isolates coupled with high genetic drift (Supplementary Table 6).

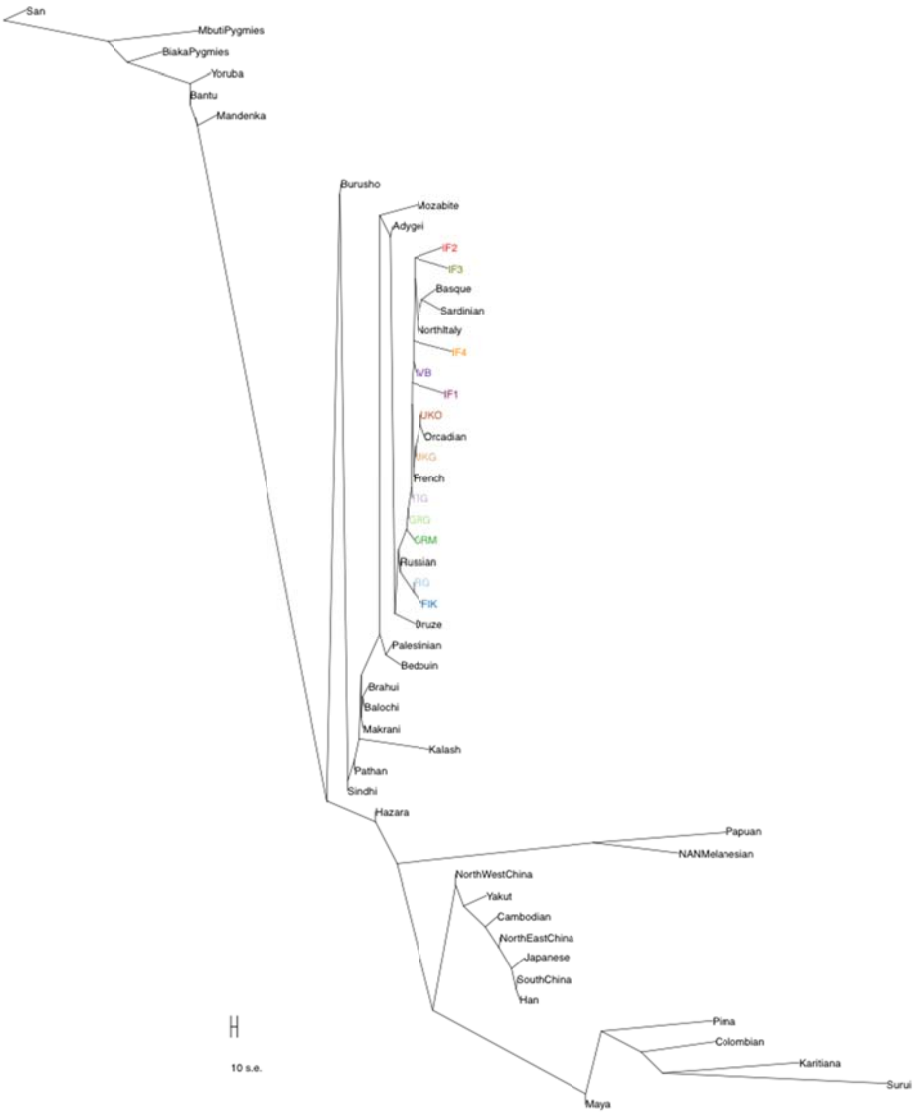

Supplementary Figure 5. Population relationships from TreeMix analysis with worldwide populations from the HGDP-CEPH panel. The analysis used the ancestry graph implemented in TreeMix v.1.12[3], using blocks of 200 SNPs to account for linkage disequilibrium, excluding SNPs with MAF <0.01 across all of the samples included here. Each isolate is close to its general population, and also close to other populations from nearby geographic locations. All isolates have longer branches than the general populations, reflecting greater genetic drift. The four north Italian isolates from Friuli Venezia Giulia (IF1, IF2, IF3 and IF4) show the longest branches among all the isolated populations.

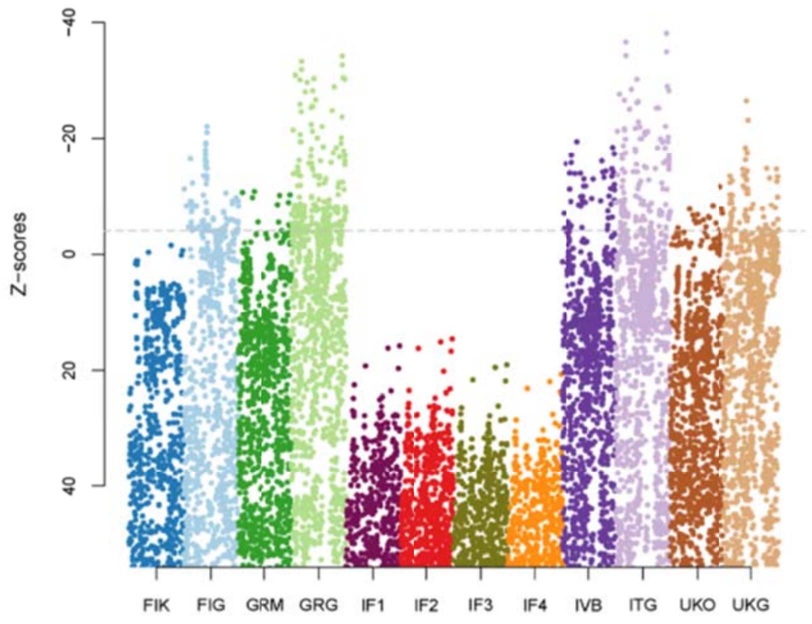

Supplementary Figure 6. Distribution of f3-statistics for each population studied. Each dot represents the f3-statistic population average value of a given population in this study compared with any two populations derived from the rest of the populations in this study and populations from the HGDP. We computed a Z-score using a block jackknife of 500 SNPs and used f3-statistics (Pop1, Pop2; X) where Pop1 and Pop2 are every possible pair of populations in our dataset plus HGDP-CEPH populations, and X is one of the populations in our dataset. The grey dotted line indicates Z scores ≤-4, and scores above the line suggest admixture between two populations. The isolates appear to have had less mixture in their history compared with the general populations. No admixture was detected in the four north Italian isolates IF1, IF2, IF3 and IF4 or FIK. We detect signals of admixture in IVB and GRM, but these remain much less than in the general populations.

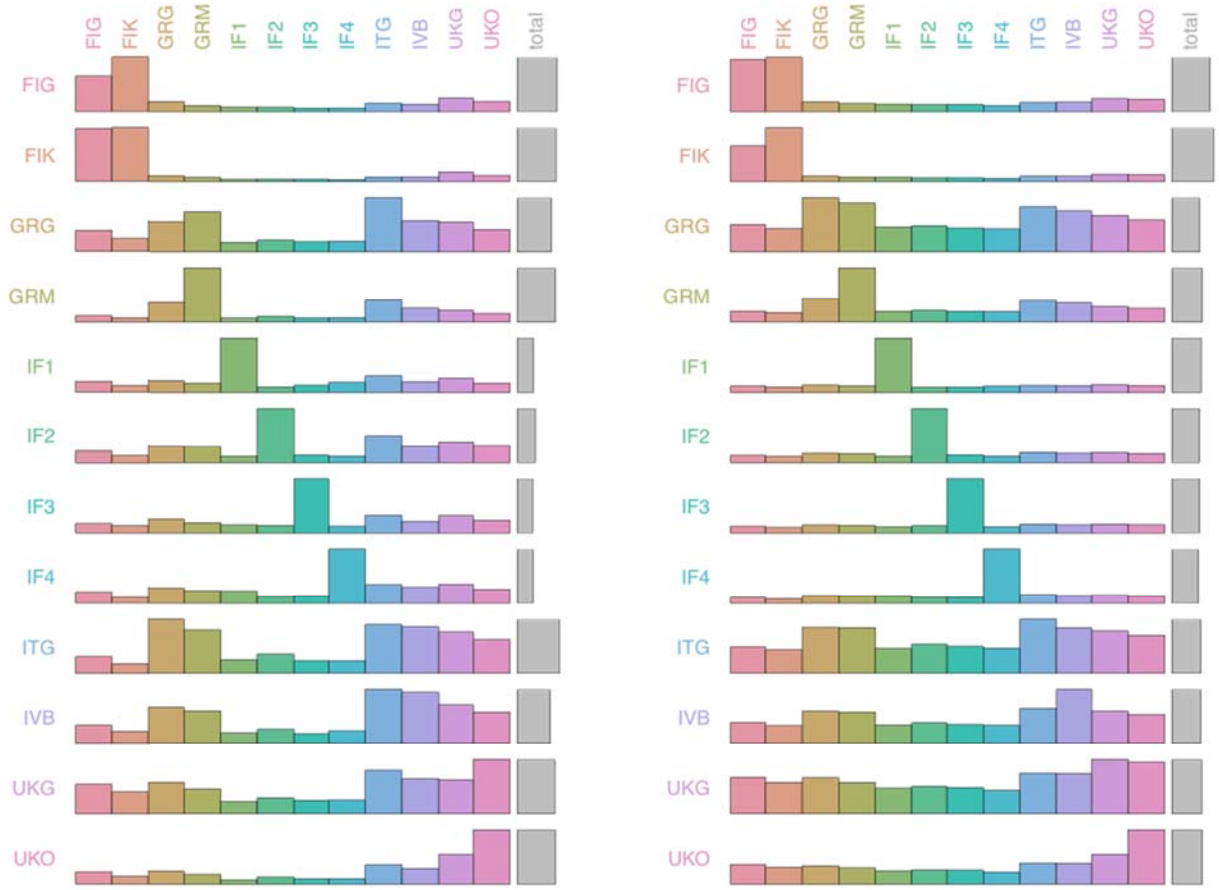

Supplementary Figure 7. Variant sharing at the population level: left, f_2 sharing; right, $f_{3\text{-}10}$ sharing. The y axis for the different color bars (except the grey one) is the proportion of the total SNPs of each row, while x axis of each grey bar is the total number of SNPs in each row. In general, each individual shared most f_2 variants with others from their own population, but sharing patterns did differ between populations. Isolates from the Friuli Venezia Giulia villages (IF1, IF2, IF3 and IF4) shared very few f_2 variants with either the closest general population (ITG) or other Friuli Venezia Giulia village populations, confirming that these isolates are indeed isolated and have had little recent admixture with any other population tested. FIK shared many f_2 variants with its closest general population (FIG), but few with other populations, so gene flow/shared ancestry within Finland contrasted with isolation from the rest of the Europe. But IVB, UKO and GRM shared more f_2 variants with both their closest general populations and the other general populations, except FIG, suggesting more extensive recent gene flow among these populations. In contrast, we see much less difference in sharing within the population and between the different populations for the $f_{3\text{-}10}$ variants, as expected. These variants are on average older than f_2 variants, and so have had more time to spread among the populations.

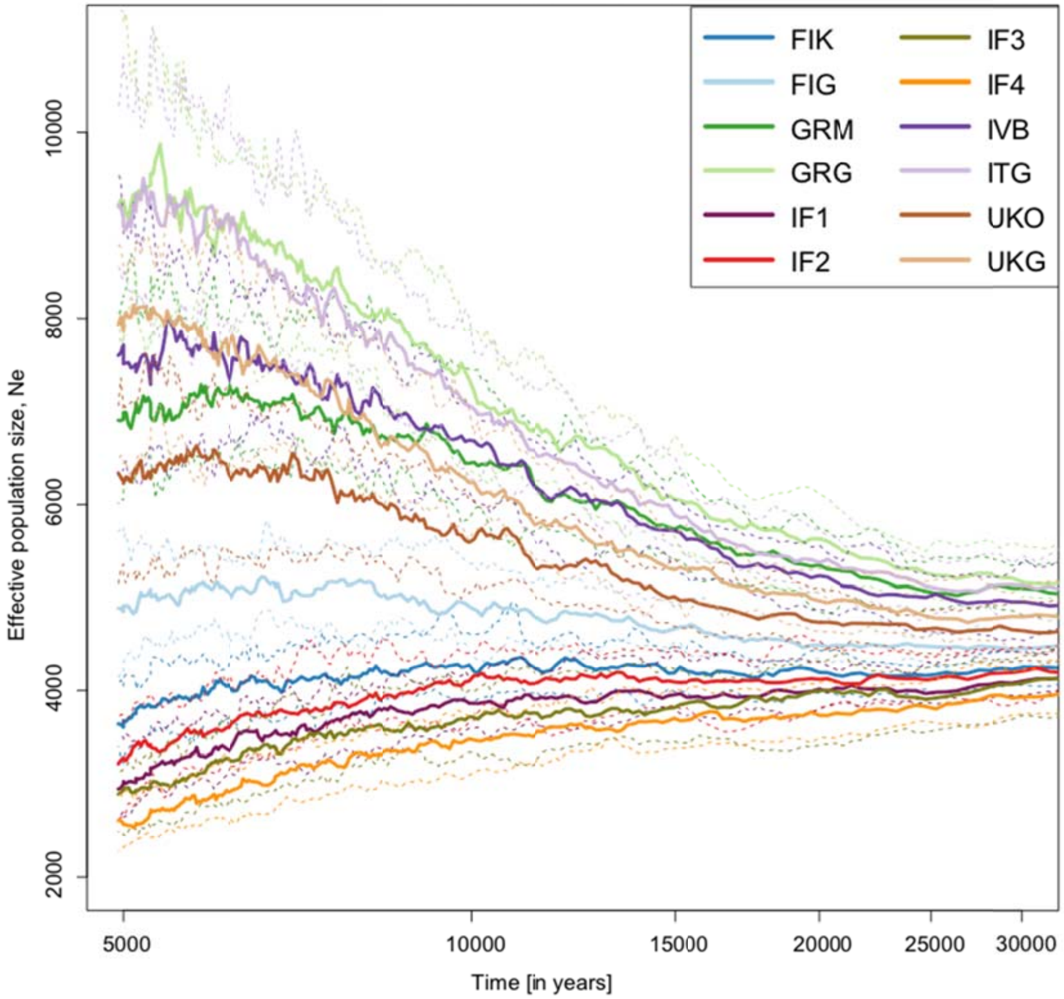

Supplementary Figure 8. LD-based demographic inference for the populations studied – long term Ne from 5,000 years ago to 30,000 thousand years ago. The x-axis shows the time in years before present. The y-axis is the average effective population size (*Ne*) at a particular time. The solid lines are medians, and the dashed lines are the 95th percentiles. Ne were estimated using LD-based methods[4-6] in the *NeON* R package[7] from the minimum dataset with common variants (MAF >5%) only. The Ne is the harmonic mean over all recombination distance classes. The median and confidence interval were estimated using the 50th, 5th and 95th percentiles of the distribution of long-term Ne given the different distance classes. The Ne estimates made here are averaged over coarse time intervals (a thousand years), yet all the isolated population have a substantially smaller Ne than their closest general population up to 10,000 year ago.

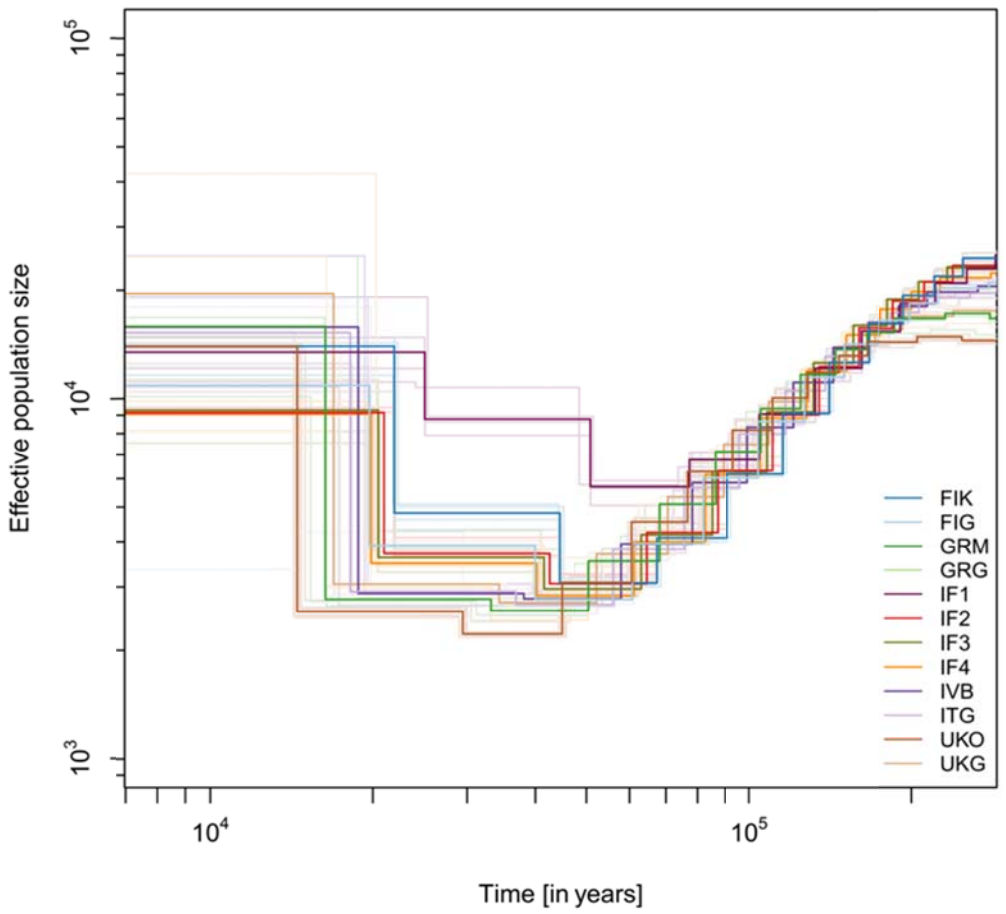

Supplementary Figure 9. Inference of effective population size history using MSMC. The Markovian coalescent (MSMC) method[8] was used to estimate the effective population size history of the populations using four individuals from each population. We accounted for low-coverage data by using a slow mutation rate of $0.8x10^{-8}$ mutations per nucleotide per generation and a longer generation time of 33 years, and ran MSMC on four genomes from every population using 40 time intervals, collecting the median from every time segment. The effective population sizes are the median of four individuals from each population estimated from MSMC. Most populations show the distinctive bottleneck of non-African populations with a minimum at ~60,000 years ago. All populations except IF1 appear to have comparable sizes before 20,000 years ago and only minor differences at later times, which are not significant.

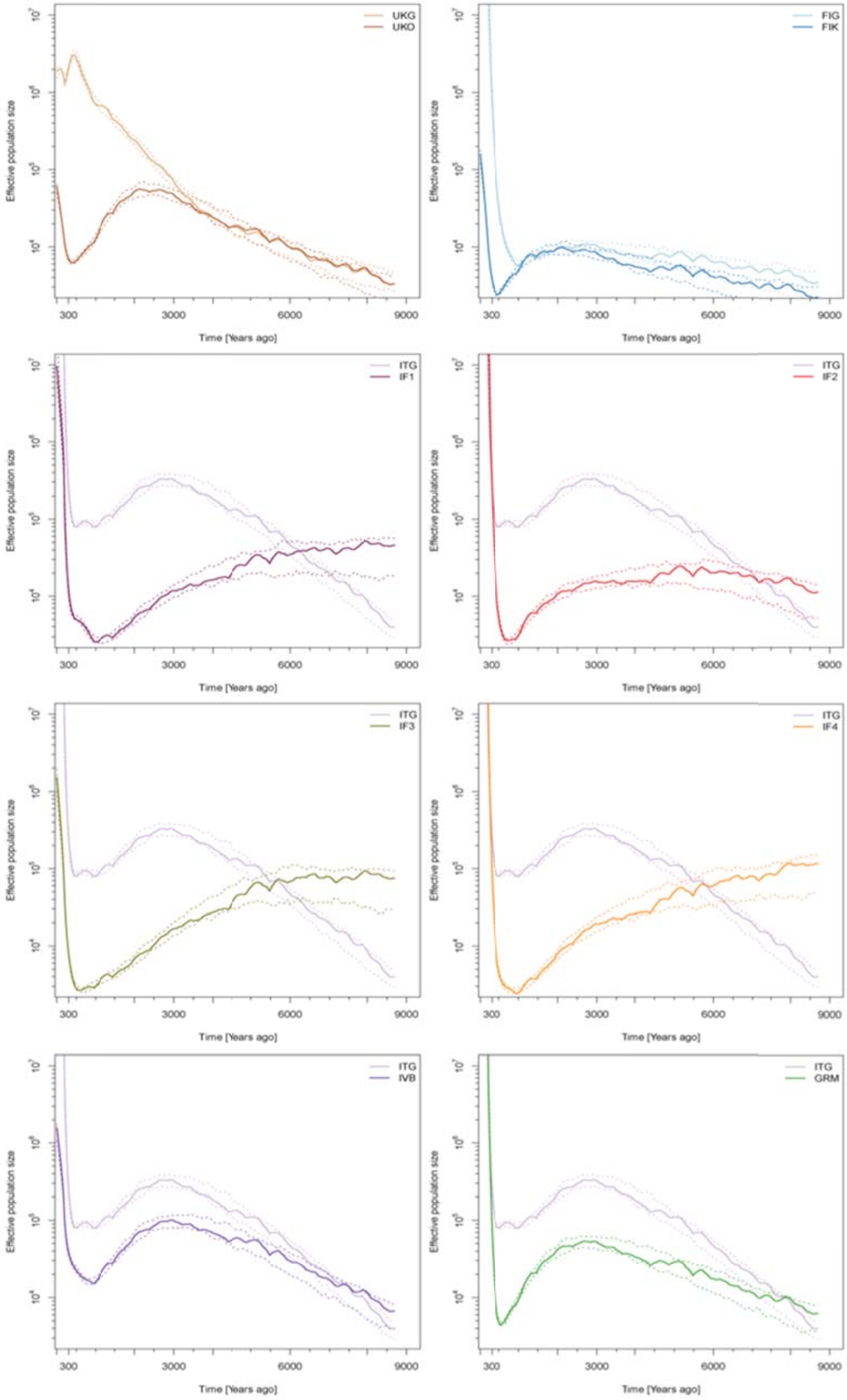
UKG
UKO
FIG
FIK
ITG
IF1
IF2
IF3
IF4
IVB
GRM
Effective population size
Time [Years ago]
300
3000
6000
9000

Supplementary Figure 10. Inference of recent population size changes using IBDNe. Population size estimates in recent times (within the last 9,000 years) were inferred from long segments of IBD using IBDNe. Isolated and general populations show contrasting patterns of population size changes in recent times. We used IBDNe[9] to estimate Ne from long segments of identity-by-descent (IBD). We used IBDseq[10] to detect IBD segments in sequence data from chromosome 2 in all populations. We then used IBDNe with the default parameters and a minimum IBD segment length of 2 centiMorgan (cM) units. We assumed a generation time of 29 years. In these analyses, we used ITG as the general population for GRM as the variant calling procedure for GRG made it unsuitable for this analysis. All general populations (except FIG) show a steady increase in size during the past 3,000 years, while the size of all isolates drops within the last 1000 years, and only recovers in the past few generations. IF1, IF2, IF3 and IF4 appear to have the sharpest decrease in population size while the IVB, on the other hand, show a drop in population size more limited in time and magnitude. Both FIK and FIG show a decrease in size; however, the FIG start increasing in the last 600 years while FIK start increasing only in the past 300 years. The UKO show a steady population size until 2,700 years ago when population size drops sharply and recovers only very recently. The GRG population seems to have been dropping in size gradually reaching the smallest size ~600 years ago before increasing in size in the past 300 years. All isolates appear to have decreased in size while the general populations (except FIG) have increased steadily in size.

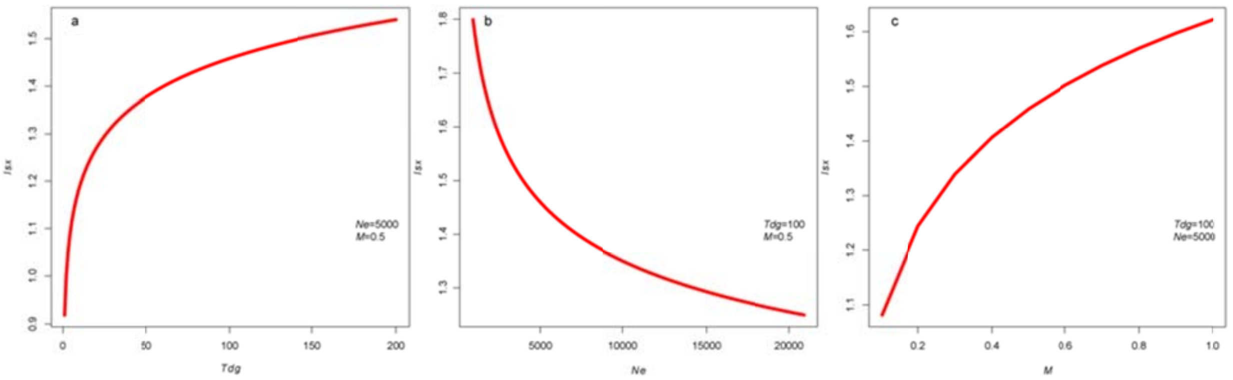

Supplementary Figure 11. The Isolation Index (*Isx*) reflects the demographic history of the isolated population. It increases with (a) deeper divergence time from the general population (Tdg), (b) smaller effective population size (*Ne*) and (c) the level of private isolate ancestry (*M*) (deduced from the level of shared ancestry with the general population).

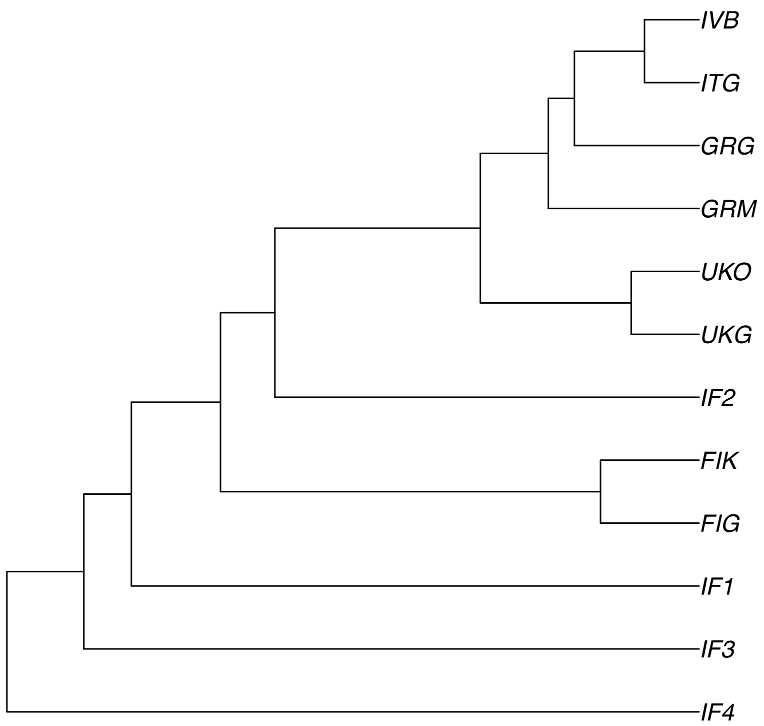

Supplementary Figure 12. UPGMA tree based on pairwise F_{ST} values between each isolate and its general population. Genome-wide F_{ST} between isolates and their general populations was calculated with the software 4P[11] using the minimum sample size dataset by removing markers in strong LD in the whole dataset and variants with MAF <0.01. A UPGMA tree based on pairwise F_{ST} was constructed using the R package *phangorn*[12]. Only IVB, FIK and UKO show a close genetic relationship with their general population, while IF1, IF2, IF3 and IF4 lie far away from their general population, which reflects the strong genetic drift in these isolates. GRM and GRG are moderately close in the tree, which might reflect intermediate divergence or calling bias.

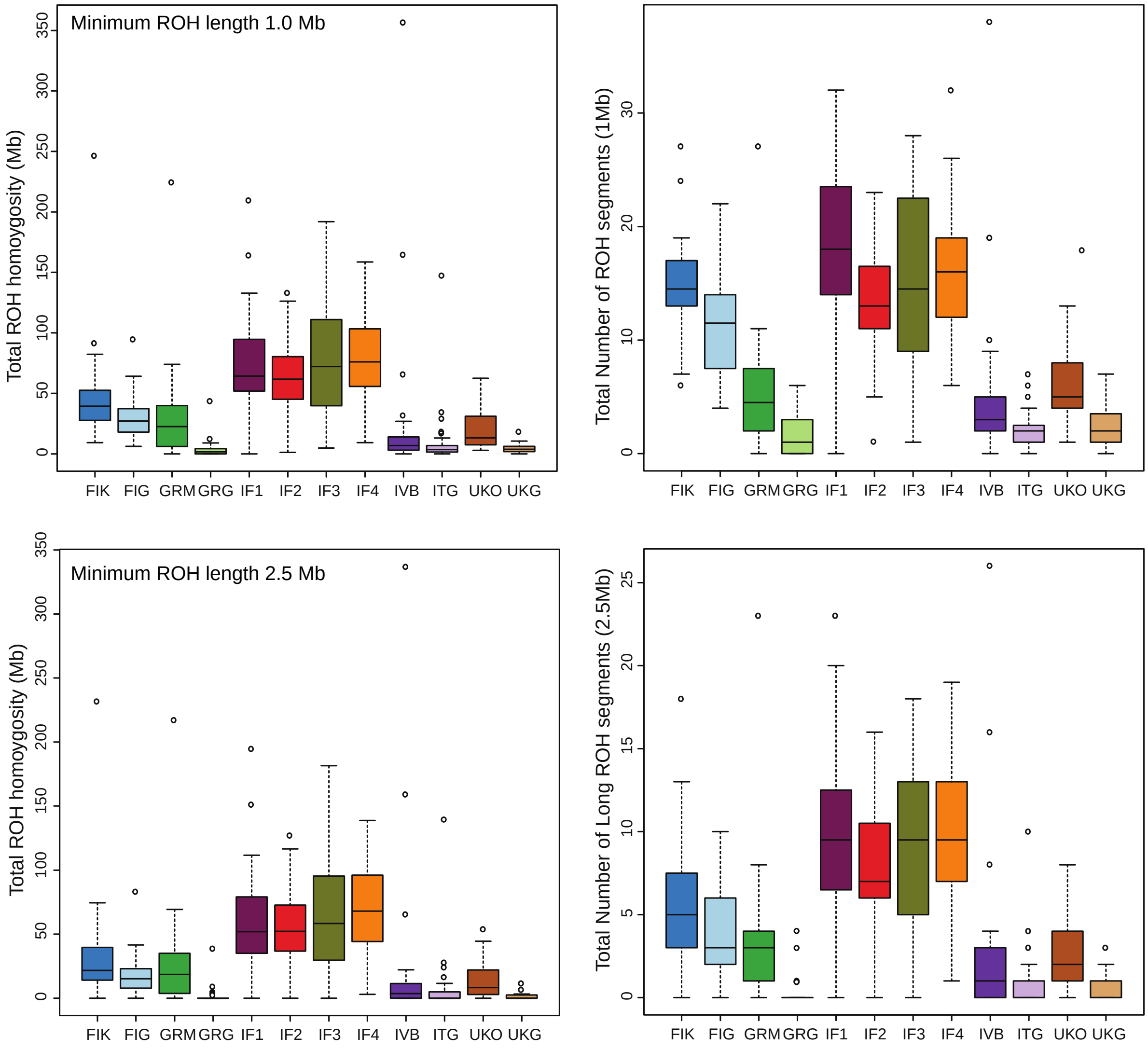

Supplementary Figure 13. Box plots of total length of ROH (left panel) and total number of ROH fragments (right panel) per individual. The top panels show ROH fragments longer than 1.0 Mb and the lower panels fragments greater than 2.5 Mb. We used the whole sample dataset but trimmed the SNPs for these analyses. The runs of homozygosity (ROHs) with a minimum length of 1.0 Mb and 2.5 Mb were calculated using PLINK[13] with LD pruning. More numbers of ROH fragments and total length of the ROH regions are seen in each isolate compared with its general population, both for ROH fragments greater than 1.0 Mb and 2.5 Mb. The four Italian isolates, IF1, IF2, IF3 and IF4, which are the most isolated, showed these characteristics most markedly, while IVB showed them the least, with FIK, GRM and UKO in between.

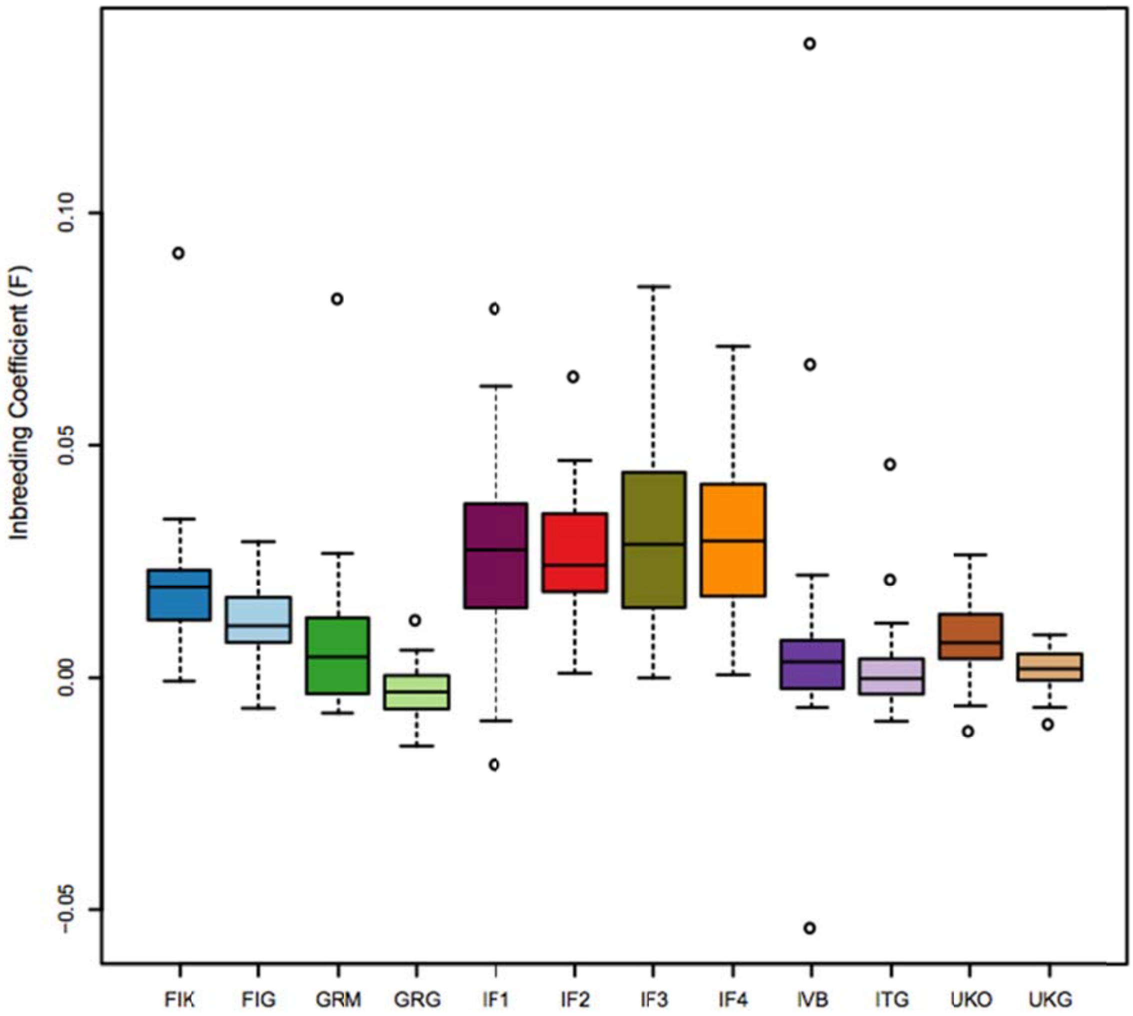

Supplementary Figure 14. Inbreeding coefficient (F) values of each individual in each population in this study. F was calculated using the LD-pruned dataset with the function het[14] implemented in PLINK. F showed a very similar pattern to ROH (Supplementary Figure 13).

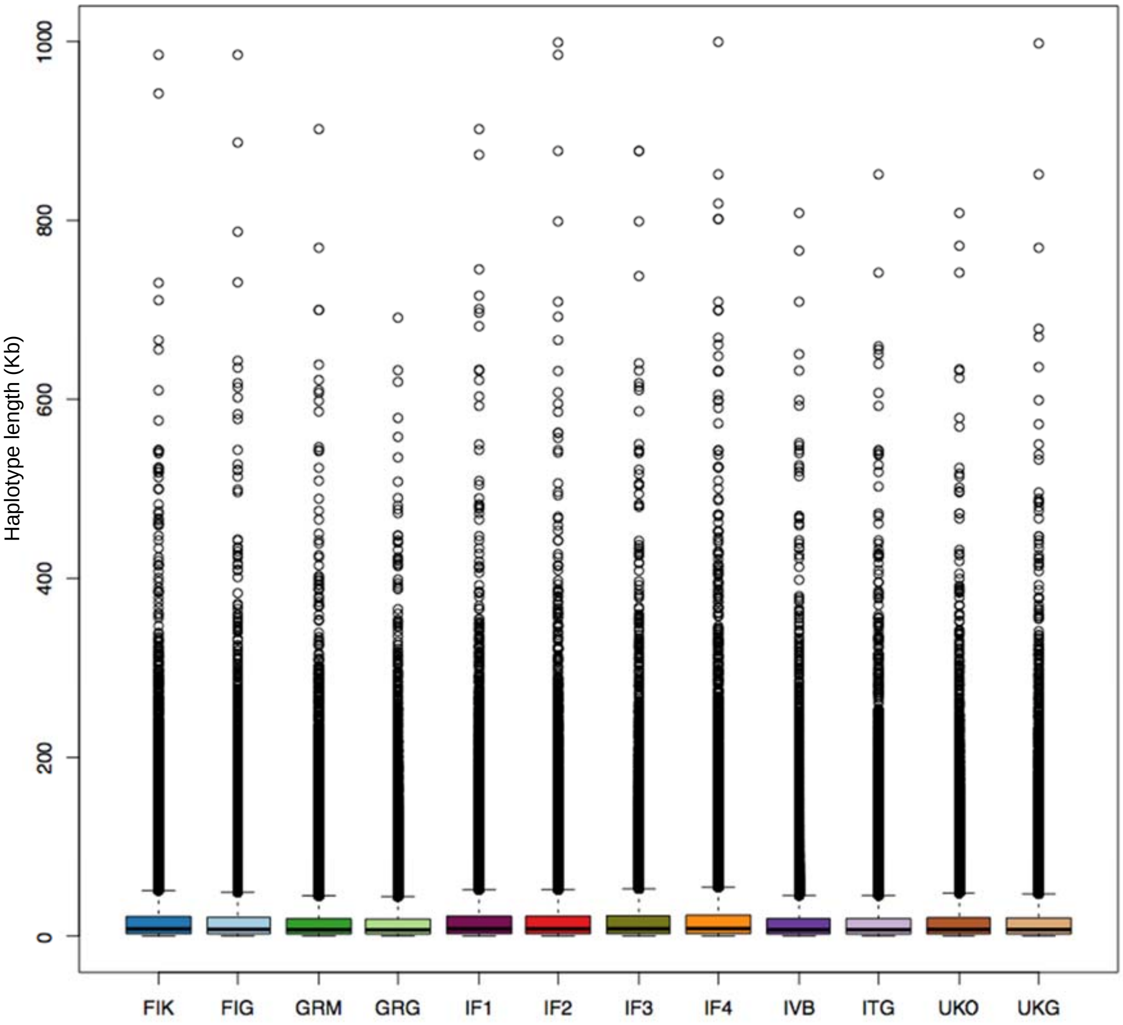

Supplementary Figure 15. Haplotype length distribution in the populations studied. The haplotype block length between variants with D' > 0.85 were estimated using PLINK[13], via Haploview's interpretation of block definition[15], using the minimum sample size dataset for this analysis, excluding variants with MAF <0.01 across all the individuals in the dataset. The average haplotype length in the isolates IF1, IF2, IF3, IF4 and FIK is significantly longer than in their general populations ITG and FIG, but no difference was observed between GRM, IVB and UKO and their general populations GRG, ITG and UKG.

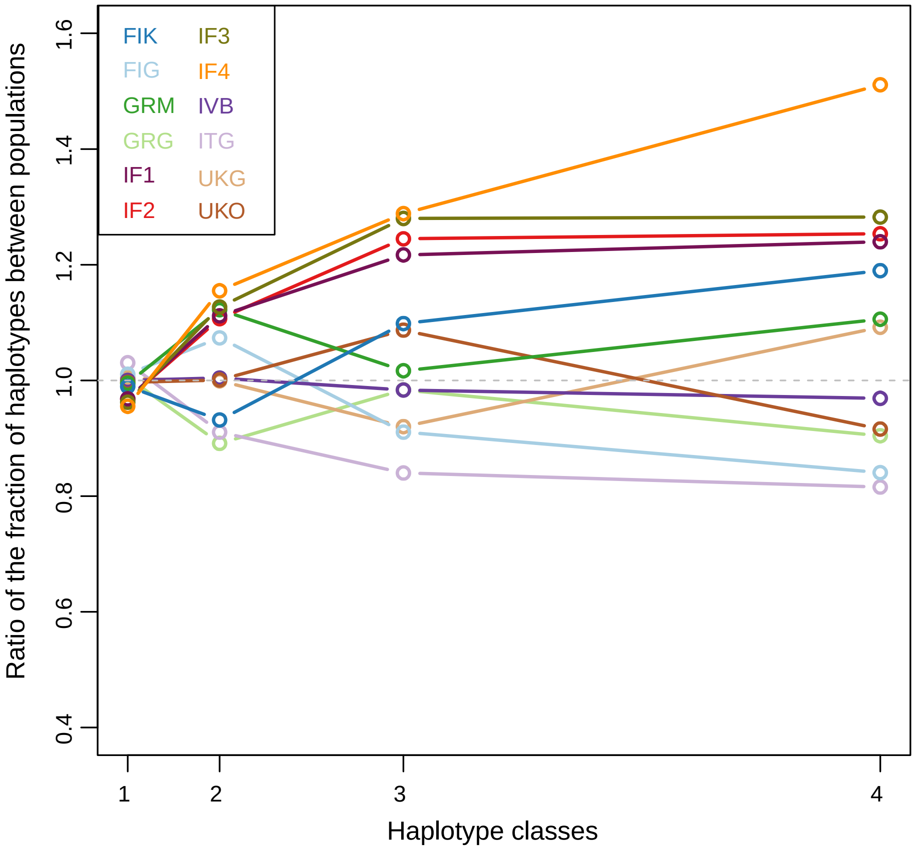

Supplementary Figure 16. The ratios in each isolate compared with its general population, or vice-versa, of haplotypes of different length classes (Supplementary Table 9) that are shared. We performed optimal k-means clustering on the distribution of the length of haplotypes using the R package Ckmeans.1d.dp[16] and divided the haplotypes into four classes (short, medium-short, medium-long and long) and the characteristics of each class are reported in Supplementary Table 9. The proportion of different classes of haplotypes in IF1, IF2, IF3, IF4 and FIK are also substantially different from their general populations. ITG and FIG, in particular, have a higher proportion of shorter haplotypes. No difference between GRM, IVB and UKO and their general populations GRG, ITG and UKG was found. These results again suggest that IF1, IF2, IF3, IF4 and FIK are more isolated that the other isolates.

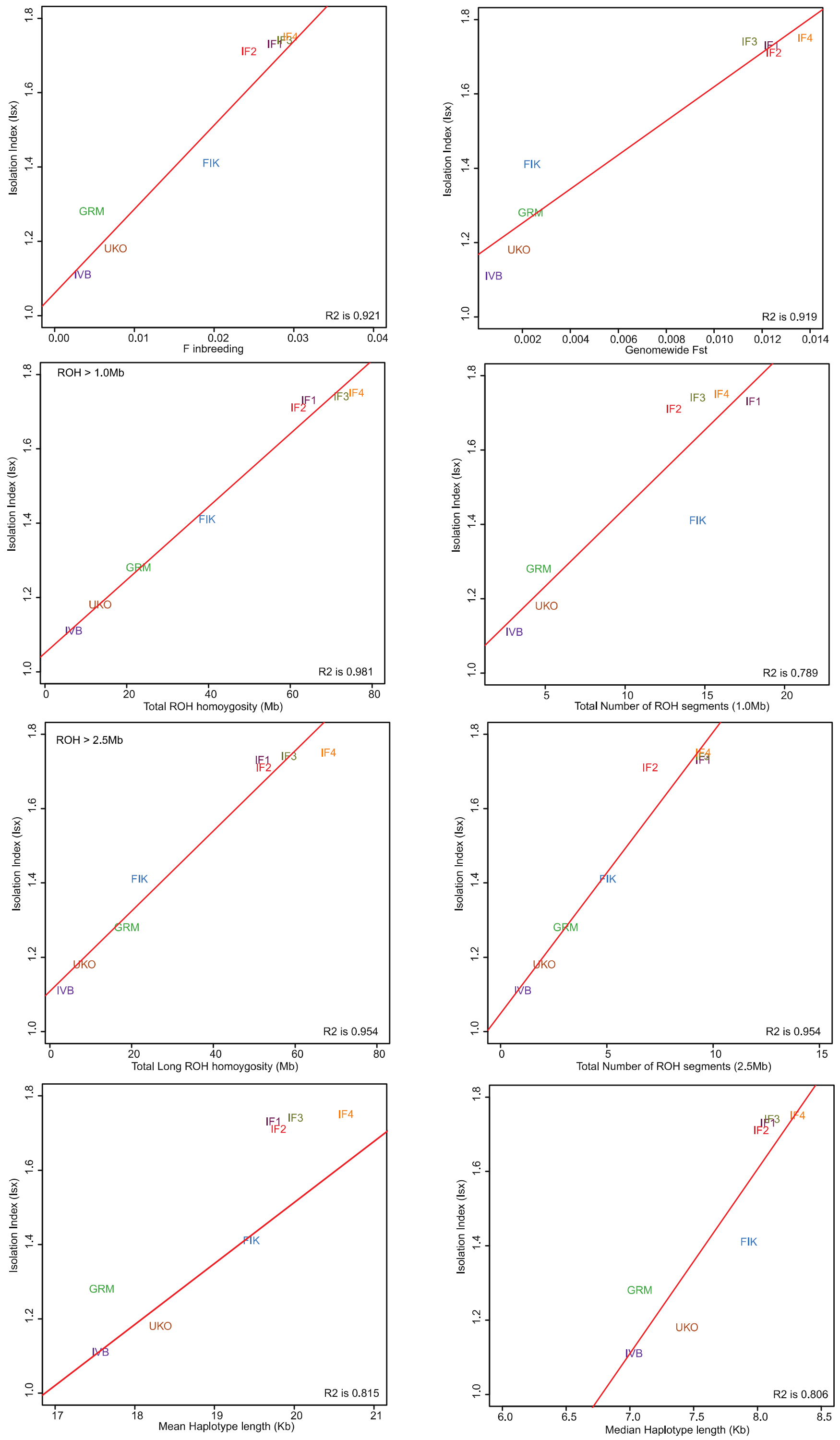

Supplementary Figure 17. Correlation plots of Isx and different measures of genetic drift. The correlation in each plot is labelled, as well as the Pearson correlation coefficient (R^2) and its p-value.

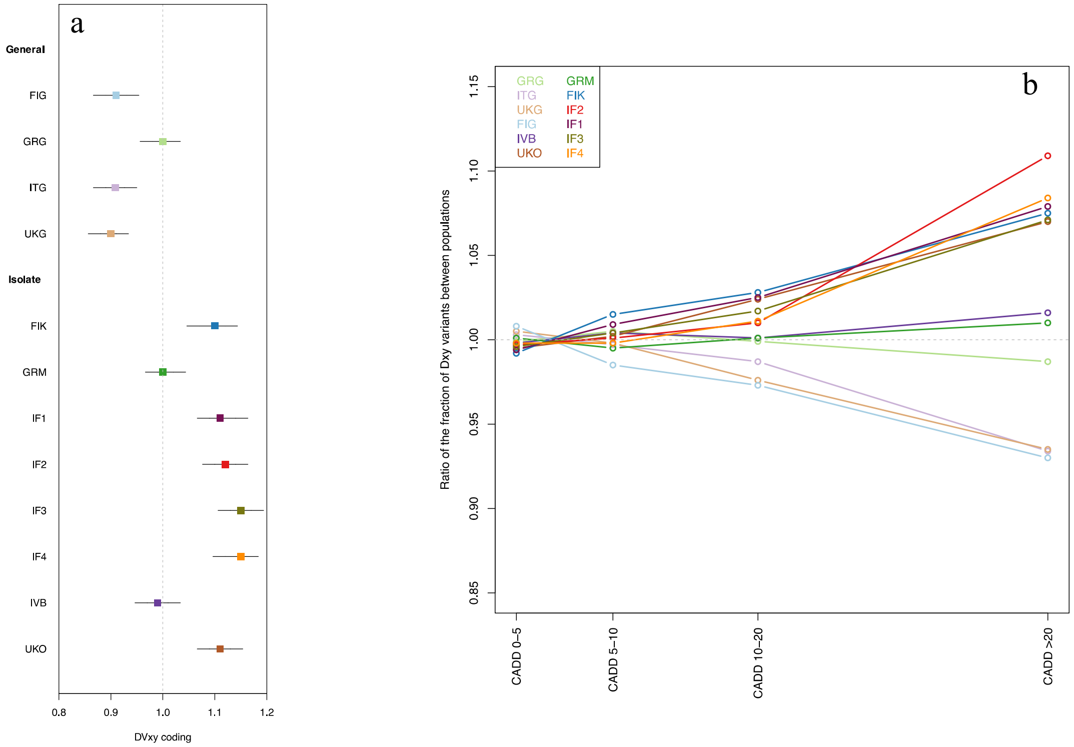

Supplementary Figure 18. *DVxy* statistics using the minimum sample size. (a) *DVxy-coding* statistic in isolates and general populations; (b) *DVxy-wg* statistics between isolates and general population in different CADD score bins.

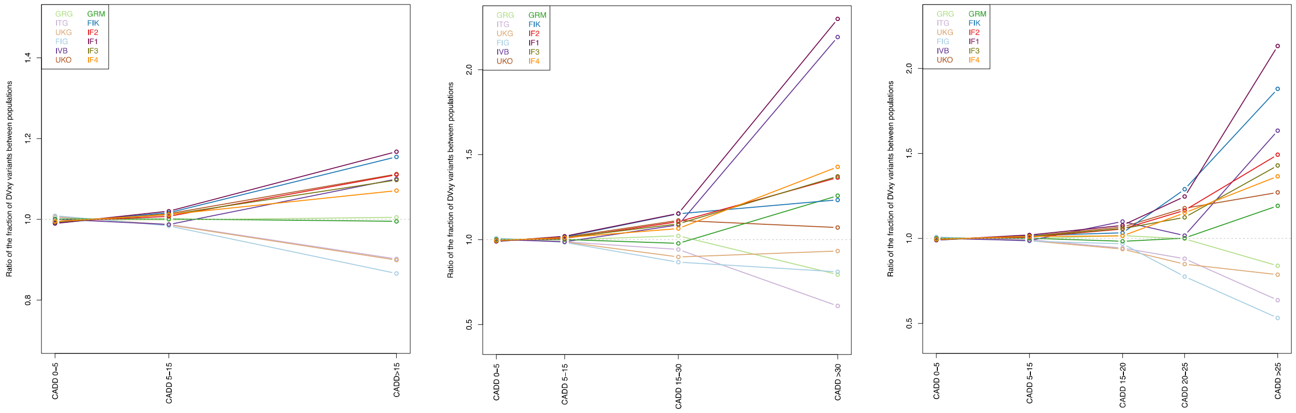

Supplementary Figure 19. *DVxy-wg* statistics in isolates and general populations, stratified by CADD score with different cut-offs and different bins.

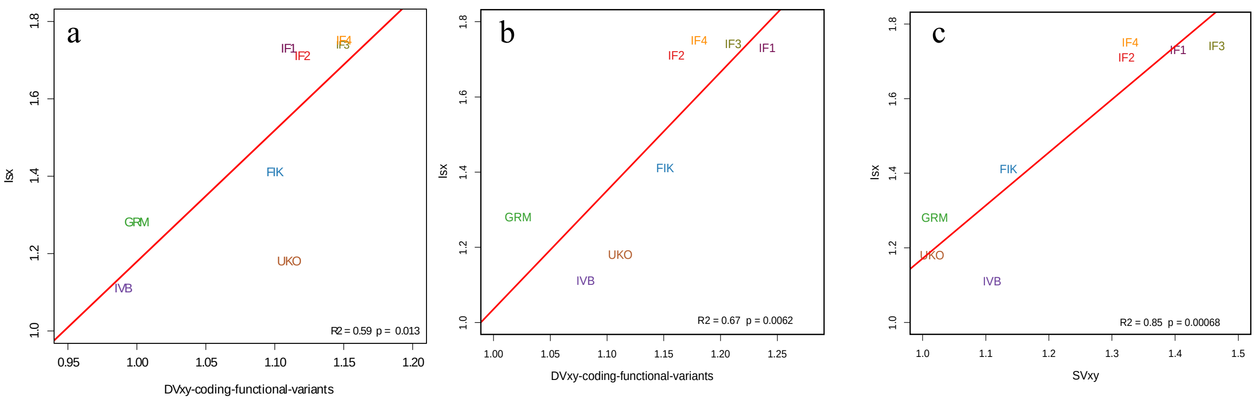

Supplementary Figure 20. Correlation between *Isx* and the *DVxy* (a. with minimum sample size. b. with matched sample size) or *SVxy* statistics (c. minimum sample size).

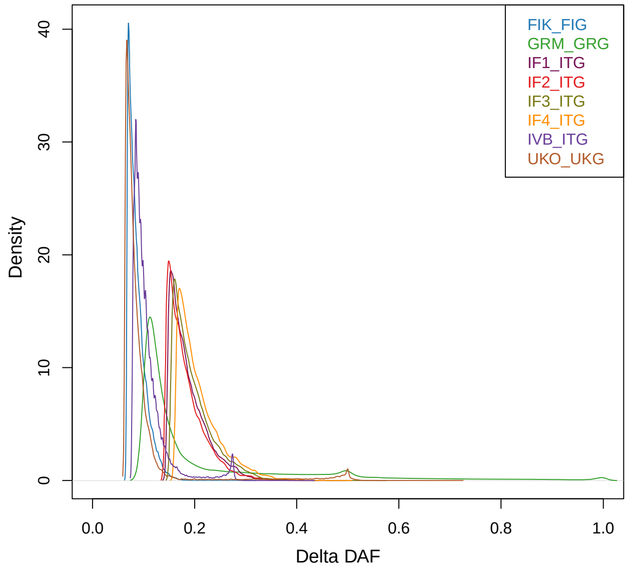

Supplementary Figure 21. 99th percentile of DAF distribution between pairs of populations. For each pair of isolate and its general population, genome-wide pairwise derived allele frequency differences (deltaDAF) were calculated as described previously[17]. The sites with extreme deltaDAF due to high DAF values in the isolate (HighD sites) were identified by scanning the genome in non-overlapping windows of 3,000 SNPs and picking the variant within each window with the highest deltaDAF value, provided that deltaDAF was above a threshold between 0.3 and 0.5. HighD sites were assigned to the population with the highest DAF in the pair.

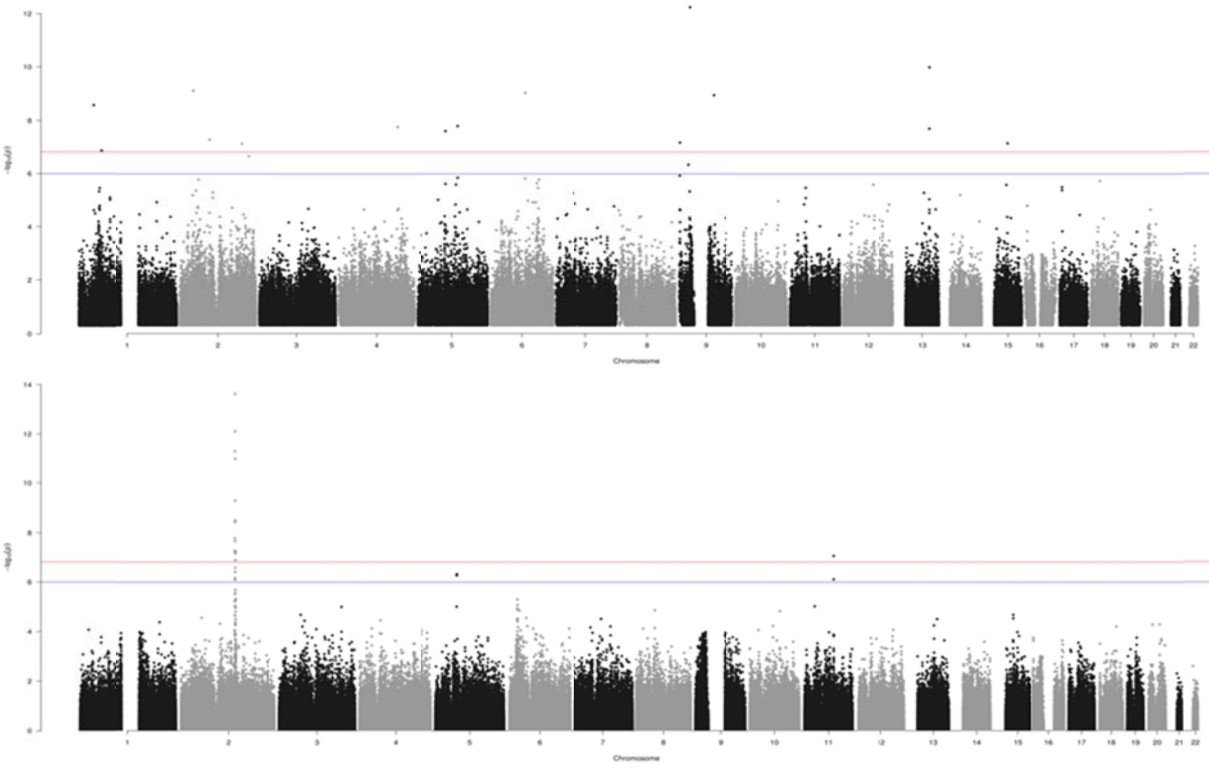

Supplementary Figure 22. Scaled SDS values in UKO (top) and UKG (bottom). The red line represents the 99.99th percentile of the distribution while the blue line genome-wide suggested significant p value. The singleton density score (SDS) analyses were performed as described [18] to one of our isolates (UKO with sample size of 397) and its general population (UKG, the UK10K data, also used in the SDS paper). We successfully replicated the selection signal at the lactose locus in the UKG, and the known selected SNP, rs4988235, is in the 99.99th percentile of the distribution. However, we failed to detect any signal at this locus in the UKO. This suggested that the sample size of UKO is too small for SDS to have power to detect even strong signals. We failed to detect any convincing extra selection signal genomewide in UKO. There are a few SNP signals with high SDS scores, but they are not clustered, and are likely false positives. As UKO has the biggest sample size and second lowest Isx value of our isolates, this suggests that we would not be able to detect any convincing positive selection signals in any of the other isolates studied here.

Supplementary Tables

Supplementary Table 1. Population and dataset information

Population (three letter name)	Sample location	Sample size	Sequence depth	Data published
Kuusamo (FIK)	Kuusamo, Finland	377	4x	No
SISu cohort (FIG)	Suomi, Finland	1564	6x	No
HELIC-MANOLIS (GRM)	Crete, Greece	249	4x	No
TEENAGE (GRG)	Athens, Greece	100	10-30x	No
Friuli Venezia Giulia (all)	Friuli Venezia Giulia, Italy	250	4-10x	No
Friuli Venezia Giulia 1 (IF1)	Friuli Venezia Giulia, Italy	60	4-10x	No
Friuli Venezia Giulia 2 (IF2)	Friuli Venezia Giulia, Italy	45	4-10x	No
Friuli Venezia Giulia 3 (IF3)	Friuli Venezia Giulia, Italy	47	4-10x	No
Friuli Venezia Giulia 4 (IF4)	Friuli Venezia Giulia, Italy	36	4-10x	No
Val Borbera (IVB)	Val Borbera, Italy	225	6x	No
Toscani (ITG)	Toscani, Italy	108	7x	Yes[19]
Orkney (UKO)	Scotland, UK	397	4x	No
UK10K (UKG)	UK (ALSPAC & TwinsUK cohorts)	3781	6.5x	Yes[20]

All of the populations have been given a three-letter abbreviation used throughout the text. The first one or two letters identify the country (FI = Finland, GR = Greece, I = Italy, UK = United Kingdom) and the last one or two letters the specific isolate (K = Kuusamo, M = MANOLIS, F = Friuli Venezia Giulia, VB = Val Borbera and O = Orkney) or the general population (G).

Samples from Friuli Venezia Giulia were collected from four different villages and were found to be genetically highly structured, so we treated them as four different isolates, and we also excluded some samples which do not genetically match any of these four groups.

Supplementary Table 2. Genotype discordance with genotype chip data, stratified by SNP class.

	REF-REF	REF-ALT	ALT-ALT
1000 Genomes	1.21%	0.11%	1.06%
Finland	0.11%	0.37%	0.30%
UK10K	0.10%	0.32%	0.27%

Supplementary Table 3. Numbers of variants in the different populations stratified by functional category. The functional annotation used the Ensembl 76 VEP pipeline with “-pick” option. ‘Novel’ variants are those not found in 1000 Genomes Project Phase 3 or UK10K project, and the proportion of novel variants is shown in brackets.

POP	predicted LoF			Nonsynon			Synon			UTR		
	MAF <=2%	MAF >2-5%	MAF >5%	MAF <=2%	MAF >2-5%	MAF >5%	MAF <=2%	MAF >2-5%	MAF >5%	MAF <=2%	MAF >2-5%	MAF >5%
FIG	2.1k (19.9%)	311 (1.0%)	967 (0.8%)	43.6k (15.8%)	6.9k (0.8%)	19.5k (0.9%)	27.9k (12.3%)	5.7k (0.7%)	20.2k (0.9%)	108.2k (12.6%)	22.5k (0.9%)	76.9k (0.8%)
FIK	1.3k (18.8%)	305 (2.0%)	971 (0.9%)	25.6k (14.6%)	7.0k (1.6%)	19.6k (0.8%)	17.0k (10.8%)	5.7k (1.2%)	20.1k (0.8%)	65.8k (11.7%)	22.7k (1.4%)	76.6k (0.8%)
GRG	917 (2.0%)	235 (0.4%)	950 (0.7%)	21.0k (2.1%)	5.4 k(0.8%)	18.5k (0.8%)	15.6k(1.8%)	4.9k (0.9%)	19.5k(0.8%)	60.2k (1.7%)	18.9k (0.7%)	74.5k (0.8%)
GRM	1.3k (9.5%)	256 (1.6%)	992 (1.0%)	3.0k (9.6%)	6.1k (1.5%)	19.6k (1.0%)	21.1k (7.1%)	5.4k (1.3%)	20.3k (0.9%)	81.3k (7.5%)	20.5k (1.2%)	77.5k (0.8%)
IF1	365 (0.8%)	283 (1.8%)	971 (1.0%)	7.6k (2.0%)	6.3k (2.3%)	19.3k (1.0%)	5.7k (1.6%)	5.3k (1.4%)	19.6 (1.0%)	22.1k (1.6%)	20.6k (1.4%)	75.2k (0.9%)
IF2	250 (2.8%)	319 (0.9%)	974 (0.6%)	5.6k (1.8%)	7.2 k(1.5%)	19.5k (0.9%)	4.2k (1.4%)	5.7k (1.1%)	20.0k (1.0%)	16.5k (1.3%)	22.2k (1.2%)	76.7k (0.8%)
IF3	244 (2.0%)	300 (0.3%)	995 (0.9%)	5.1k (1.8%)	6.7k (1.8%)	20.0k (1.0%)	3.8 (1.5%)	5.3k (1.6%)	20.5k (0.9%)	14.7k (1.5%)	21.1k (1.2%)	78.0k (0.9%)
IF4	227 (0.4%)	238 (0.4%)	977 (1.2%)	5.2k (2.1%)	5.1k (1.6%)	19.7k (1.1%)	4.0k (1.5%)	4.2k (1.1%)	20.1k (1.0%)	15.8k (1.5%)	16.2k (1.3%)	76.9k (0.9%)
IVB	1.2k (2.1%)	259 (0.0%)	999 (1.2%)	28.1k (2.3%)	6.0k (1.1%)	19.6k (0.9%)	20.0k (2.0%)	5.2k (0.8%)	20.3k (0.9%)	77.6k (1.8%)	19.8k (0.8%)	77.2k (0.8%)
UKO	1.9k (21.7%)	284 (0.4%)	981 (1.2%)	39.3 (16.0%)	6.4 k(0.9%)	19.5k (1.0%)	25.1k (12.3%)	5.3k (0.7%)	20.2k (0.9%)	99.0k (12.9%)	21.2k (0.8%)	76.6k (0.8%)
**Total**	**4.6k (23.0%)**	**1.2k (1.0%)**	**1.4k (0.6%)**	**94.2k (17.9%)**	**26.8k (1.2%)**	**29.1k (0.6%)**	**60.8k (13.5%)**	**21.6k (0.8%)**	**28.0k (0.4%)**	**236.1k (14.3%)**	**83.9k (0.7%)**	**107.0k (0.3%)**

POP	Intron			Regulatory			Intergenic		
	MAF <=2%	MAF >2-5%	MAF >5%	MAF <=2%	MAF >2-5%	MAF >5%	MAF <=2%	MAF >2-5%	MAF >5%
FIG	3.4m (11.9%)	777k (0.8%)	3.0m (0.7%)	634k(12.4%)	142k (1.0%)	542k (0.8%)	2.4m (11.6%)	580k (0.8%)	2.3m (0.7%)
FIK	2.1m (11.0%)	788k (1.2%)	3.0m (0.7%)	389k (11.0%)	144k (1.3%)	539k (0.8%)	1.5m (10.7%)	581k (1.1%)	2.3m (0.7%)
GRG	1.9m (1.6%)	667k (0.7%)	3.0m (0.7%)	357k (1.7%)	122k (0.7%)	524k (0.8%)	1.4m (1.5%)	500k (0.7%)	2.3m (0.7%)
GRM	2.6m (7.3%)	728k (1.2%)	3.0m (0.8%)	490k (7.6%)	133k (1.2%)	547k (0.9%)	1.9m (7.2%)	549k (1.1%)	2.4m (0.8%)
IF1	742k (1.4%)	720k (1.3%)	2.9m (0.8%)	137k (1.4%)	131k (1.3%)	527k (0.9%)	540 (1.3%)	535k (1.2%)	2.3m (0.8%)
IF2	543k (1.3%)	790k (1.0%)	3.0m (0.8%)	100k (1.4%)	143k (1.1%)	538k (0.8%)	393k (1.2%)	578k (1.0%)	2.3m (0.7%)
IF3	491k6 (1.3%)	735k (1.1%)	3.0m (0.8%)	90.5k (1.3%)	135k (1.2%)	545k (0.9%)	353k (1.2%)	546k (1.0%)	2.4m (0.7%)
IF4	526k (1.3%)	569k (1.1%)	3.0m (0.8%)	97.8k (1.4%)	105k (1.1%)	538k (0.9%)	381k (1.2%)	419k (1.0%)	2.3m (0.8%)
IVB	2.5m (1.7%)	703k (0.8%)	3.0m(0.8%)	462k (1.8%)	128k (0.8%)	548k (0.9%)	1.8m (1.6%)	527k (0.8%)	2.4m (0.8%)
UKO	3.1m (11.9%)	748k (0.8%)	3.0m (0.8%)	573k (12.0%)	136k (0.8%)	541k (0.9%)	2.2m (11.4%)	550k (0.7%)	2.3m (0.8%)
**Total**	**7.3m (13.8%)**	**2.9m (0.7%)**	**4.0m (0.3%)**	**1.4m (13.9%)**	**530.7k (0.8%)**	**732.8k (0.4%)**	**5.2m (13.3%)**	**2.1m (0.7%)**	**3.1m (0.3%)**

The numbers and proportions of rare variants (MAF ≤2%) in each population that are novel are very different, as expected because of the different sample sizes. In contrast, they are not very different for both variants with MAF >2-≤5% and common variants (>5%). It is striking that we still found ~10-20 thousand novel variants with MAF >2-≤5% and 40-50 thousand novel common (MAF >5%) variants even after a comparison with the 1000 Genomes Project Phase 3 and UK10K project. The counts for different functional categories show the same pattern but the proportions of novel rare variants in important functional categories, such as predicted LoF and non-synonymous, are significantly higher than the other variants due to the strength of purifying selection. In total, 4551, 1206 and 1409 unique predicted LoF variants and 94,221, 26,838 and 29,121 non-synonymous variants are found in the MAF bins of ≤2%, >2-≤5 %, and >5% among the 10 newly-sequenced populations here: 23.0%, 1.0% and 0.6% for predicted LoFs and 17.9%, 1.2% and 0.6% for non-synonymous were novel. In all, we find more than 1.4 thousand novel common predicted LoF variants and more than 29 thousand novel non-synonymous ones.

Supplementary Table 4. Numbers and proportions (percentage) of deleterious variants that have drifted to high frequency in the isolates compared with all four general populations studied here

Isolates	Total	Missense plus LoF	CADD score 15
**FIK**	70,579	410 (0.58%)	1479 (2.1%)
**GRM**	49,884	266 (0.53%)	988 (2.0%)
**IF1**	119,157	689 (0.58%)	2676 (2.2%)
**IF2**	94,496	518 (0.55%)	2080 (2.2%)
**IF3**	107,281	616 (0.57%)	2417 (2.3%)
**IF4**	122,254	688 (0.56%)	2792 (2.3%)
**IVB**	30,284	154 (0.51%)	530 (1.8%)
**UKO**	36,512	210 (0.58%)	634 (1.7%)

Supplementary Table 5. Median numbers of variant sites, homozygous sites, heterozygous sites and alleles per genome. The functional annotation used Ensembl 76 VEP pipeline with the "-pick" option. Numbers to the left-hand side of the grey line are based on the minimum-sample-size and those on the right-hand side are based on matched-sample-size. hom = homozygous, het = heterozygous.

POP	Counts	Total	Singletons	predicted LoF	Nonsynon	Synon	UTR	Intron	Regulatory	Intergenic	Total	Singletons	predicted LoF	Nonsynon	Synon	UTR	Intron	Regulatory	Intergenic
**FIK**	Site	3.647m	6.265k	227	9.804k	11.689k	31.308k	1.768m	88.437k	1.328m	3.760m	559	579	11.535k	12.478k	24.595k	1.913m	136.576k	1.369m
	hom	1.675m	-	82	4.169k	5.260k	14.034k	809.203k	40.428k	612.828k	1.793m	-	260	5.199k	5.934k	11.469k	910.943k	64.626k	654.291k
	het	1.973m	6.265k	146	5.629k	6.423k	17.268k	959.066k	48.037k	715.095k	1.966m	559	318	6.337k	6.546k	13.132k	1.002m	71.985k	714.229k
	allele	5.323m	6.265k	310	13.974k	16.928k	45.329k	2.577m	128.864k	1.941m	5.553m	559	839	16.732k	18.41k	36.067k	2.824m	201.229k	2.023m
**FIG**	Site	3.653m	7.390k	232	9.792k	11.713k	31.374k	1.769m	88.697k	1.331m	3.763m	1.707k	581	11.533k	12.522k	24.627k	1.915m	136.789k	1.370m
	hom	1.665m	-	87	4.171k	5.218k	14.008k	806.442k	40.131k	608.952k	1.786m	-	260	5.169k	5.903k	11.419k	907.312k	64.277k	651.696k
	het	1.987m	7.390k	144	5.598k	6.493k	17.372k	962.268k	48.592k	722.667k	1.979m	1.707k	319	6.364k	6.613k	13.209k	1.008m	72.549k	718.277k
	allele	5.318m	7.390k	322	13.957k	16.944k	45.380k	2.575m	128.793k	1.939m	5.550m	1.707k	840	16.705k	18.423k	36.045k	2.8227m	201.127k	2.021m
**GRM**	Site	3.661m	7.601k	233	9.815k	11.691k	31.340k	1.772m	88.972k	1.333m	3.775m	2.761k	580	11.539k	12.539k	24.619k	1.920m	137.420k	1.374m
	hom	1.649m	-	85	4.085k	5.166k	13.841k	797.349k	39.709k	602.955k	1.767m	-	255	5.090k	5.8255k	11.288k	897.665k	63.485k	644.612k
	het	2.012m	7.601k	146	5.747k	6.541k	17.512k	975.361k	49.304k	729.964k	2.009m	2.761k	326	6.456k	6.720k	13.316k	1.022m	73.959k	729.399k
	allele	5.309m	7.601k	316	13.900k	16.847k	45.219k	2.569m	128.696k	1.936m	5.541m	2.761k	835	16.635k	18.359k	35.925k	2.818m	200.883k	2.019m
**GRG**	Site	3.613m	5.357k	227	9.684k	11.595k	31.060k	1.750m	87.661k	1.317m	3.732m	1.668k	573	11.383k	12.421k	24.420k	1.898m	135.581k	1.359m
	hom	1.683m	-	86	4.186k	5.231k	14.060k	815.443k	40.350k	614.883k	1.783m	-	253	5.152k	5.850k	11.335k	907.572k	64.057k	650.132k
	het	1.931m	5.357k	140	5.480k	6.3615k	16.955k	935.528k	47.324k	701.329k	1.949m	1.668k	319	6.217k	6.571k	13.114k	990.794k	71.548k	708.973k
	allele	5.297m	5.357k	313	13.866k	16.846k	45.156k	2.565m	127.975k	1.931m	5.516m	1.668k	828	16.526k	18.276k	35.749k	2.806m	199.646k	2.010m
**IF1**	Site	3.622m	1.632k	227	9.680k	11.596k	31.087k	1.754m	88.006k	1.318m	3.738m	318	579	11.394k	12.416k	24.418k	1.903m	135.763k	1.360m
	hom	1.688m	-	88	4.212k	5.311k	14.166k	816.281k	40.613k	617.404k	1.802m	-	263	5.207k	5.958k	11.485k	915.587m	64.810k	658.126k
	het	1.934m	1.632k	138	5.463k	6.272k	16.929k	938.394k	47.418k	700.840k	1.937m	318	317	6.191k	6.457k	12.923k	986.896m	71.056k	702.143k
	allele	5.309m	1.632k	315	13.899k	16.886k	45.243k	2.571m	128.467k	1.936m	5.541m	318	841	16.618k	18.380k	35.893k	2.818m	200.634k	2.018m
**IF2**	Site	3.625m	1.847k	221	9.691k	11.563k	31.140k	1.757m	87.986k	1.319m	3.739m	486	571	11.417k	12.395k	24.454k	1.904m	135.859k	1.360m
	hom	1.679m	-	88	4.189k	5.247k	13.987k	810.867k	40.285k	614.363k	1.797m	-	261	5.215k	5.913k	11.445k	911.918k	64.589k	655.477k
	het	1.947m	1.847k	135	5.484k	6.333k	17.123k	946.908k	47.738k	705.242k	1.943m	486	309	6.210k	6.473k	13.020k	991.794k	71.306k	705.661k
	allele	5.305m	1.847k	311	13.864k	16.837k	45.128k	2.568m	128.269k	1.934m	5.536m	486	830	16.604k	18.314k	35.884k	2.816m	200.361k	2.016m
**IF3**	Site	3.627m	1.520k	226	9.677k	11.577k	31.148k	1.757m	87.829k	1.320m	3.741m	397	574	11.373k	12.396k	24.399k	1.904m	135.817k	1.361m
	hom	1.681m	-	86	4.163k	5.269k	14.036k	812.099k	40.567k	613.980k	1.799m	-	262	5.178k	5.942k	11.462k	913.511m	64.874k	656.27k
	het	1.947m	1.520k	140	5.513k	6.326k	17.088k	945.889k	47.283k	706.701k	1.942m	397	314	6.203k	6.453k	12.914k	990.697m	70.979k	704.284k
	allele	5.306m	1.520k	312	13.840k	16.852k	45.196k	2.569m	128.468k	1.935m	5.537m	397	838	16.555k	18.338k	35.858k	2.816m	200.592k	2.017m
**IF4**	Site	3.625m	1.543k	223	9.677k	11.613k	31.174k	1.756m	87.868k	1.319m	3.739m	502	570	11.406k	12.434k	24.471k	1.903m	135.800k	1.360m
	hom	1.682m	-	87	4.199k	5.279k	14.109k	813.289k	40.589k	615.793k	1.799m	-	260	5.204k	5.933k	11.438k	914.520k	64.735k	657.570k
	het	1.943m	1.543k	135	5.496k	6.367k	17.064k	942.591k	47.219k	702.757k	1.940m	502	311	6.223k	6.521k	13.019k	987.802k	71.062k	701.724k
	allele	5.306m	1.543k	309	13.882k	16.876k	45.260k	2.568m	128.476k	1.935m	5.537m	502	828	16.621k	18.355k	35.913k	2.816m	200.637k	2.017m
**IVB**	Site	3.654m	4.941k	231	9.773k	11.708k	31.376k	1.769m	88.666k	1.332m	3.766m	1.309k	577	11.496k	12.517k	24.615k	1.916m	136.937k	1.371m
	hom	1.653m	-	86	4.101k	5.1801k	13.828k	800.205k	39.766k	604.670k	1.771m	-	255	5.112k	5.840k	11.305k	9003.975m	63.583k	646.898k
	het	2.001m	4.941k	146	5.669k	6.524k	17.503k	968.987k	49.003k	727.829k	1.994m	1.309k	322	6.387k	6.681k	13.305k	1.015m	73.363k	724.014k
	allele	5.309m	4.941k	317	13.885k	16.883k	45.227k	2.569m	128.428k	1.936m	5.539m	1.309k	834	16.615k	18.345k	35.904k	2.816m	200.638k	2.018m
**ITG**	Site	3.671m	21.23k	232	9.901k	11.768k	31.600k	1.778m	89.115k	1.336m	3.784m	16.387k	585	11.649k	12.579k	24.779k	1.926m	137.521k	1.376m
	hom	1.650m	-	85	4.107k	5.176k	13.778k	797.200k	39.726k	602.671k	1.768m	-	254	5.104k	5.832k	11.262k	897.714k	63.548k	645.640k
	het	2.020m	21.23k	146	5.796k	6.590k	17.820k	981.517k	49.337k	733.736k	2.016m	16.387k	330	6.533k	6.761k	13.490k	1.029m	73.941k	730.493k
	allele	5.322m	21.23k	315	14.03k	16.930k	45.388k	2.576m	128.751k	1.939m	5.552m	16.387k	837	16.746k	18.414k	36.056k	2.825m	201.043k	2.022m
**UKO**	Site	3.663m	10.777k	238	9.877k	11.700k	31.463k	1.774m	88.932k	1.335m	3.777m	2.926k	587	11.611k	12.552k	24.684k	1.921m	137.475k	1.375m
	hom	1.662m	-	84	4.131k	5.200k	13.941k	803.908k	40.010k	606.834k	1.780m	-	257	5.134k	5.875k	11.385k	904.697m	63.924k	649.107k
	het	2.000m	10.777k	153	5.781k	6.496k	17.485k	970.318k	48.942k	728.286k	1.997m	2.926k	329	6.482k	6.671k	13.309k	1.016m	73.548k	725.565k
	allele	5.326m	10.777k	322	13.989k	16.926k	45.378k	2.578m	128.968k	1.941m	5.556m	2.926k	844	16.740k	18.426k	36.044k	2.825m	201.399k	2.024m
**UKG**	Site	3.655m	7.087k	226	9.743k	11.667k	31.362k	1.770m	88.606k	1.332m	3.767m	2.129k	582	11.497k	12.489k	24.594k	1.917m	136.904k	1.372m
	hom	1.657m	-	84	4.150k	5.218k	13.874k	802.008k	39.906k	604.127k	1.776m	-	258	5.137k	5.872k	11.313k	902.597k	63.876k	647.237k
	het	1.997m	7.087k	142	5.593k	6.442k	17.418k	968.229k	48.569k	728.079k	1.992m	2.129k	325	6.357k	6.617k	13.287k	1.015m	73.046k	724.702k
	allele	5.312m	7.087k	310	13.890k	16.874k	45.211k	2.572m	128.514k	1.936m	5.543m	2.129k	839	16.626k	18.365k	35.934k	2.819m	200.761k	2.019m

Supplementary Table 6. FKi statistics.

Population	Median (FKi)	Maximum (FKi)
FIK/FIG	-0.014	0.618
GRM/GRG	-0.025	0.256
IF1/ITG	-0.045	0.882
IF2/ITG	-0.036	0.804
IF3/ITG	-0.041	0.851
IF4/ITG	-0.031	0.834
IVB/ITG	-0.003	0.026
UKO/UKG	-0.023	0.252

FKi median and maximum values for each isolate compared with its general population, using K=8 in the ADMIXTRUE analysis

Supplementary Table 7. Divergence time of each isolate from its closest general population estimated using the LD-based method.

Isolate	Time of divergence from the closest general population (generations)	CI (5th - 95th percentile)
FIK	26	25-28
GRM	40	38-44
IF1	154	144-164
IF2	137	127-146
IF3	159	148-170
IF4	176	166-188
IVB	18	17-19
UKO	21	18-22

The divergence times estimated from LD have large uncertainties, but we see that FIK, GRM and IVB diverged from their closest general population more recently than the four north Italian isolates, IF1, IF2, IF3 and IF4. In particular, the divergence time of FIK from FIG around 26 generations ago (750 years) fits the historical divergence time of mid-16th century.

Supplementary Table 8. Demographic parameters and *Isx* values for each isolate

Isolated population	*Tdg* in generations (5-95th percentile)	*M*	Long-term *Ne* (5-95th percentile)	*Isx* (5-95th percentile)
FIK	26 (25-28)	0.71	4226 (3972-4399)	1.41(1.39-1.42)
GRM	40 (38-44)	0.42	6242 (6011-6907)	1.28 (1.25-1.30)
IF1	154 (144-164)	0.99	3806 (3529-4075)	1.73 (1.70-1.75)
IF2	137 (127-146)	0.99	3960 (3595-4271)	1.71(1.68-1.73)
IF3	159 (148-170)	0.99	3656 (3289-3939)	1.74 (1.71-1.77)
IF4	176 (166-188)	0.91	3390 (3158-3634)	1.75 (1.72-1.77)
IVB	18 (17-19)	0.31	6439 (5955-6711)	1.11 (1.09-1.12)
UKO	21(18-22)	0.37	5592 (5248-5990)	1.18 (1.15-1.20)

As each isolate has a different demographic history, isolation levels are different. IF1, IF2, IF3 and IF4 are the most isolated populations with the highest Isx values, while IVB is the least isolated one with the lowest Isx. The highest Isx values reflect a combination of smaller Ne, longer isolation time and lower migration between the isolate and its general population.

Supplementary Table 9. Characteristics of each haplotype class.

Class	Description	Mean (kb)	Standard Deviation (kb)
1	short	7.1	6.6
2	medium-short	44.1	14.8
3	medium-long	118.9	32.3
4	long	310.2	116.2

Supplementary Table 10. Summary of haplotype features in the populations studied.

Population	Total N. haplotype	Mean length (kb)	Mann-Whitney p-value	Median (kb)	5th-95th percentile (kb)	Fraction of class 1 haplotype	Fraction of class 2 haplotype	Fraction of class 3 haplotype	Fraction of class 4 haplotype
FIK	49,693	19.45	< 0.0001	7.92	0.33-75.81	0.7826	0.1579	0.0390	0.0048
FIG	49,643	18.57	n.a.	7.51	0.30-72.16	0.7910	0.1696	0.0355	0.0040
GRM	48,771	17.61	0.009117	7.07	0.31-68.62	0.8054	0.1736	0.0331	0.0036
GRG	48,661	17.12	n.a	6.83	0.29-67.83	0.8095	0.1547	0.0325	0.0032
IF1	49,986	19.73	< 0.0001	8.07	0.34-77.26	0.7790	0.1764	0.0399	0.0047
IF2	49,851	19.79	< 0.0001	8.02	0.32-77.64	0.7788	0.1756	0.0408	0.0048
IF3	49,957	20.01	< 0.0001	8.11	0.33-78.42	0.7744	0.1788	0.0419	0.0049
IF4	49,657	20.64	< 0.0001	8.31	0.35-79.69	0.7688	0.1833	0.0422	0.0058
IVB	48,570	17.55	0.5179	7.02	0.30-69.16	0.8048	0.1593	0.0322	0.0037
ITG	48,371	17.63	n.a	7.03	0.29-68.69	0.8048	0.1587	0.0327	0.0038
UKO	49,295	18.03	0.02151	7.44	0.31-72-34	0.7952	0.1649	0.0363	0.0035
UKG	49,062	17.98	n.a	7.26	0.30-69.72	0.7979	0.1648	0.0334	0.0038

The average haplotype length in the isolates IF1, IF2, IF3, IF4 and FIK is significantly longer than in their general populations ITG and FIG, but no difference was observed between GRM, IVB and UKO and their general populations GRG, ITG and UKG. The proportion of different classes of haplotypes in IF1, IF2, IF3, IF4 and FIK are also substantially different from in their general populations: ITG and FIG, in particular, have a higher proportion of shorter haplotypes. No difference between GRM, IVB and UKO and their general populations GRG, ITG and UKG was found. These results again suggest that IF1, IF2, IF3, IF4 and FIK are more isolated that the other isolates.

Supplementary Table 11. Pairwise correlation coefficients (Person's correlation coefficient, r)

	Isx	F_{ST}	F	ROH (1.0 Mb)	Haplotype -length	*Dvxy-coding*
F_{ST}	0.975					
F	0.969	0.901				
ROH (1.0 Mb)	0.992	0.948	0.955			
Haplotype-length	0.918	0.866	0.977	0.941		
Dvxy-coding	0.801	0.772	0.859	0.787	0.848	
SVxy	0.912	0.901	0.905	0.920	0.929	0.72

Supplementary Table 12. *Rxy* statistics.

Population pair	*Rxy - missense*	*Rxy -LoF*	*Rxy* -variants with CADD>10	*Rxy* -variants with CADD>20
FIK-FIG	1.007 (1.002-1.013)	0.991 (0.967-1.014)	1.000 (0.999-1.000)	0.998 (0.995-1.002)
GRM-GRG	1.017 (1.005-1.029)	1.047 (0.993-1.102)	1.011 (1.010-1.013)	1.011 (1.005-1.016)
GRM-ITG	-	-	1.000 (0.999-1.002)	0.996 (0.991-1.000)
IF1- ITG:60	0.983 (0.972-0.995)	1.005 (0.954-1.057)	0.995 (0.993-0.997)	0.988 (0.981-0.995)
IF2- ITG:45	0.989 (0.978-1.000)	0.963 (0.910-1.016)	0.993 (0.991-0.995)	0.985 (0.978-0.992)
IF3- ITG:47	0.984 (0.973-0.996)	0.979 (0.926-1.032)	0.995 (0.993-0.997)	0.988 (0.980-0.995)
IF4- ITG:36	0.982 (0.969-0.995)	0.966 (0.910-1.022)	0.992 (0.990-0.995)	0.988 (0.980-0.997)
IVB-ITG	0.971 (0.717-1.227)	1.000 (0.967-1.033)	0.993 (0.992-0.994)	0.986 (0.982-0.990)
UKO-UKG	1.009 (1.003-1.016)	1.019 (0.984-1.055)	1.003 (1.002-1.004)	1.004 (1.001-1.008)

Overall, we did not find any isolate that showed a significantly higher genetic burden for either *Rxy-missense* or *Rxy-LoF* variants, although we see a marginally lower genetic burden for missense variants in IF1, IF3 and IF4. *Rxy* using variants with CADD scores greater than 10 and 20 should increase statistical power, since we include a larger set of genome-wide functional variants. We also failed to find convincing evidence to support higher or lower genetic loads in the isolates. These results are consistent with previous studies, as the genetic load is affected by both population demography and selection[21].

Supplementary Table 13. *DVxy-coding* statistics in each population. The value at the top of each cell is the median and below in brackets are the 95th percentiles. Coding DVs are missense plus LoF variants.

Population	No. of missense DVs	No. of coding DVs	No. of intergenic DVs	*DVx-missense*	*DVx-coding*	*DVxy - missense*	*DVxy – coding*
FIK	694<br>(657-735)	724<br>(663-772)	42662<br>(39870-45600)	1.27<br>(1.25-1.30)	1.28<br>(1.25-1.32)	1.14<br>(1.10-1.20)	1.14<br>(1.10-1.20)
FIG	610<br>(553-644)	626<br>(573-667)	42459<br>(38294-45351)	1.12<br>(1.07-1.15)	1.13<br>(1.08-1.17)	0.88<br>(0.83-0.91)	0.87<br>(0.83-0.91)
GRM	1053<br>(984-1111)	1084<br>(1014-1153)	75156<br>(70312-79977)	1.20<br>(1.17-1.22)	1.2<br>(1.17-1.21)	1.02<br>(1-1.05)	1.02<br>(0.99-1.04)
GRG	1176<br>(1091-1242)	1200<br>(1116-1254)	84491<br>(80696-90914)	1.18<br>(1.15-1.20)	1.18<br>(1.15-1.2)	0.98<br>(0.96-1.00)	0.98<br>(0.96-1.01)
IF1	2020<br>(1666-2113)	2086<br>(1726-2170)	123238<br>(108383-131977)	1.33<br>(1.3-1.35)	1.31<br>(1.29-1.33)	1.24<br>(1.22-1.27)	1.24<br>(1.21-1.27)
IF2	2345<br>(1969-2461)	2414<br>(2053-2528)	139062<br>(121300-147611)	1.29<br>(1.27-1.3)	1.29<br>(1.28-1.30)	1.17<br>(1.15-1.18)	1.16<br>(1.15-1.18)
IF3	1868<br>(1596-1961)	1926<br>(1607-2019)	109618<br>(94308-116039)	1.32<br>(1.31-1.34)	1.32<br>(1.31-1.34)	1.21<br>(1.2-1.23)	1.22<br>(1.20-1.24)
IF4	1600<br>(1344-1666)	1655<br>(1392-1740)	99244<br>(87929-105109)	1.28<br>(1.26-1.3)	1.28<br>(1.26-1.3)	1.18<br>(1.16-1.21)	1.18<br>(1.16-1.21)
IVB	814<br>(743-862)	850<br>(784-897)	52765<br>(48339-55892)	1.29<br>(1.27-1.32)	1.31<br>(1.29-1.33)	1.06<br>(1.03-1.09)	1.08<br>(1.05-1.12)
ITG_IF1	1903<br>(1786-2004)	1948<br>(1850-2039)	159276<br>(145544-168162)	1.07<br>(1.06-1.09)	1.08<br>(1.06-1.09)	0.81<br>(0.79-0.82)	0.81<br>(0.79-0.82)
ITG_IF2	2784<br>(2645-2938)	2860<br>(2755-3038)	209247<br>(192533-222310)	1.10<br>(1.10-1.12)	1.11<br>(1.1-1.12)	0.86<br>(0.85-0.87)	0.86<br>(0.85-0.87)
ITG_IF3	2696<br>(2555-2825)	2768<br>(2607-2910)	201718<br>(189391-213989)	1.09<br>(1.08-1.10)	1.09<br>(1.08-1.10)	0.82<br>(0.81-0.83)	0.82<br>(0.81-0.83)
ITG_IF4	2198<br>(2072-2305)	2240<br>(2112-2360)	169550<br>(158619-182002)	1.08<br>(1.06-1.09)	1.08<br>(1.06-1.10)	0.85<br>(0.83-0.86)	0.85<br>(0.83-0.86)
ITG_IVB	672<br>(609-700)	674<br>(614-717)	47913<br>(44727-51476)	1.21<br>(1.19-1.25)	1.21<br>(1.18-1.23)	0.94<br>(0.91-0.97)	0.93<br>(0.89-0.95)
UKO	482<br>(436-506)	486<br>(440-516)	30663<br>(28650-32864)	1.28<br>(1.24-1.31)	1.29<br>(1.27-1.33)	1.14<br>(1.09-1.19)	1.11<br>(1.07-1.17)
UKG	383<br>(340-401)	398<br>(349-419)	28584<br>(26464-30568)	1.13<br>(1.08-1.16)	1.15<br>(1.1-1.18)	0.88<br>(0.84-0.92)	0.90<br>(0.85-0.94)

Supplementary Table 14. *DVxy-wg* statistics for each population. The value at the top of each cell is the median and below in brackets are the 95th percentiles.

**Populations**	**%. of DV CADD 0-5**	**% of DV CADD 5-10**	**%. of DV CADD 10-20**	**%. of DV CADD >20**	***DVxy CADD 0-5***	***DVxy CADD 5-10***	***DVxy CADD 10-20***	***DVxy CADD >20***
FIK	76.59 (76.3-76.83)	15.62 (15.52-15.75)	6.68 (6.58-6.82)	1.10 (1.05-1.15)	0.991 (0.986-0.998)	1.014 (0.999-1.035)	1.021 (0.987-1.048)	1.387 (1.266-1.509)
FIG	77.36 (77.12-77.48)	15.36 (15.24-15.52)	6.55 (6.41-6.67)	0.81 (0.76-0.85)	1.009 (1.002-1.014)	0.986 (0.966-1.001)	0.979 (0.954-1.013)	0.721 (0.663-0.79)
GRM	77.67 (77.49-77.84)	15.37 (15.27-15.46)	6.18 (6.10-6.23)	0.81 (0.78-0.84)	1.000 (0.997-1.005)	1.008 (0.996-1.017)	1.025 (1.005-1.052)	1.081 (0.996-1.121)
GRG	77.39 (77.17-77.54)	15.39 (15.27-15.51)	6.03 (6.02-6.06)	0.75 (0.73-0.79)	1.000 (0.995-1.003)	0.992 (0.983-1.004)	0.975 (0.95-0.995)	0.925 (0.901-1.017)
IF1	76.54 (76.35-76.81)	15.76 (15.63-15.85)	6.7 (6.58-6.77)	1.00 (0.96-1.03)	0.989 (0.985-0.995)	1.012 (0.998-1.022)	1.058 (1.028-1.081)	1.354 (1.261-1.439)
IF2	76.71 (76.5-76.92)	15.65 (15.48-15.76)	6.66 (6.60-6.72)	1.01 (0.96-1.04)	0.994 (0.991-1.000)	1.006 (0.987-1.014)	1.027 (1.004-1.049)	1.211 (1.154-1.295)
IF3	76.77 (76.49-77.08)	15.53 (15.35-15.65)	6.74 (6.61-6.82)	0.99 (0.95-1.04)	0.993 (0.990-0.998)	1.004 (0.993-1.014)	1.045 (1.019-1.066)	1.166 (1.089-1.27)
IF4	76.78 (76.65-77.26)	15.68 (15.36-15.77)	6.56 (6.45-6.67)	0.95 (0.92-0.98)	0.994 (0.990-1.001)	1.011 (0.989-1.022)	1.022 (0.994-1.048)	1.180 (1.120-1.267)
IVB	77.18 (76.97-77.42)	15.42 (15.27-15.52)	6.49 (6.40-6.58)	0.92 (0.87-0.98)	1.000 (0.997-1.005)	0.976 (0.959-0.986)	1.045 (1.016-1.077)	1.095 (1.018-1.191)
ITG_IF1	77.33 (77.13-77.44)	15.57 (15.48-15.71)	6.34 (6.30-6.42)	0.74 (0.72-0.77)	1.011 (1.005-1.015)	0.989 (0.978-1.002)	0.974 (0.954-0.996)	0.825 (0.772-0.866)
ITG_IF2	77.19 (77.07-77.28)	15.53 (15.5-15.58)	6.46 (6.38-6.55)	0.82 (0.78-0.84)	1.006 (1.000-1.009)	0.994 (0.986-1.014)	0.945 (0.925-0.973)	0.739 (0.695-0.793)
ITG_IF3	77.2 (76.97-77.31)	15.48 (15.44-15.58)	6.48 (6.41-6.56)	0.86 (0.83-0.89)	1.007 (1.002-1.010)	0.996 (0.987-1.007)	0.957 (0.938-0.981)	0.858 (0.787-0.918)
ITG_IF4	77.07 (76.86-77.36)	15.58 (15.42-15.68)	6.51 (6.39-6.61)	0.82 (0.79-0.85)	1.006 (0.999-1.011)	0.989 (0.979-1.011)	0.978 (0.955-1.006)	0.848 (0.789-0.893)
ITG_IVB	77.3 (77.08-77.4)	15.77 (15.64-15.87)	6.15 (6.11-6.30)	0.81 (0.79-0.84)	1.000 (0.995-1.003)	1.025 (1.014-1.043)	0.957 (0.929-0.984)	0.914 (0.840-0.982)
UKO	76.79 (76.54-77.07)	15.75 (15.57-15.89)	6.49 (6.41-6.65)	0.94 (0.87-1.01)	0.993 (0.989-0.997)	1.019 (1.006-1.034)	1.016 (0.991-1.035)	1.205 (1.089-1.294)
UKG	77.38 (77.14-77.63)	15.45 (15.22-15.59)	6.38 (6.30-6.55)	0.78 (0.74-0.82)	1.007 (1.003-1.011)	0.981 (0.967-0.994)	0.984 (0.966-1.009)	0.830 (0.773-0.918)

Supplementary Table 15. G_{SV} and *SVxy* statistics in each pair of populations.

Population pairs	Isx	Total number of genes	Total number of essentail genes	Total number of non-essentail genes	percentage of the essential genes with SV>1 in isolates	percentage of the non-essential genes with SV>1 in isolates	percentage of the essential genes with SV>1 in general population	percentage of the non-essential genes with SV>1 in general population	Gsv_isolates	Gsv_general population	Mean_SVxy	SD_SVxy
FIK_FIG	1.41	2957	271	2609	0.76	0.75	0.66	0.72	1.01	0.92	1.134	0.061
GRM_GRG	1.28	3131	308	2823	0.75	0.71	0.69	0.71	1.06	0.97	1.016	0.063
IF1_ITG	1.73	2473	231	2242	0.79	0.77	0.68	0.72	1.03	0.94	1.403	0.103
IF2_ITG	1.71	2757	264	2493	0.78	0.76	0.68	0.72	1.03	0.94	1.321	0.085
IF3_ITG	1.74	2624	251	2373	0.82	0.78	0.64	0.71	1.05	0.90	1.464	0.114
IF4_ITG	1.75	2550	253	2297	0.72	0.77	0.69	0.71	0.94	0.97	1.327	0.085
IVB_ITG	1.11	3953	387	3566	0.72	0.72	0.68	0.71	1.00	0.96	1.108	0.062
UKO_UKG	1.18	3540	357	3183	0.72	0.73	0.67	0.72	0.99	0.93	1.012	0.049

The isolates showed a higher proportion of essential genes with SV >1 relative to non-essential ones, compared with their general populations. The distribution of Gsv scores in the isolates is significantly different from the scores in the general populations (Mann-Whitney U test, p value = 0.0039) with relatively higher values of Gsv in the isolates. The SVxy statistics are significantly greater than 1 for FIG and four Italian isolates, IF1, IF2, IF3 and IF4, but not for GRM, IVB and UGO, which could be due to the separate calling method for the GRG and sample ascertainment for all three. Overall, both *Gsv* and *SVxy* statistics suggest a relaxation of purifying selection in the isolates.

Supplementary Table 16. Summary of numbers of highly differentiated sites in the isolates.

	DeltaDAF ≥0.5	DeltaDAF ≥0.4	DeltaDAF ≥0.3	PCadapt outlier with deltaDAF ≥0.5	Pcadapt outlier with deltaDAF ≥0.4	Pcadapt outlier with deltaDAF ≥0.3
FIG-FIK	0	0	1	0	0	0
GRG-GRM	0	0	0	0	0	0
ITG-IF1	6	28	52	0	23	119
ITG-IF2	1	17	52	0	16	204
ITG-IF3	4	36	54	0	26	249
ITG-IF4	6	49	54	0	57	516
ITG-IVB	3	8	22	0	0	0
UKG-UKO	35	45	52	0	4	6

We identified in total 47, 170 and 249 unique HighD sites in the eight isolates with deltaDAF greater than or equal to 0.5, 0.4 and 0.3, respectively. We did not find any sites in the FIK with deltaDAF greater than 0.5 and only one site with deltaDAF greater than 0.3, which reflects the recent divergence from FIG. The UKO showed the highest number of HighD sites with deltaDAF ≥ 0.5. However, of the sites with deltaDAF ≥ 0.5, 42 of 47 lie in segmental duplication regions, or other repeat regions, which are likely artifacts. However, one of the other five is the well-known lactose tolerance SNP (rs4988183) in IF1 compared with ITG. IF1's ancestral population is from north Europe, so this is likely to represent a site selected between north and south European populations, rather than IF1-specific selection. We failed to find compelling biological evidence for positive selection at the other four sites.

Supplementary Table 17. Overlap between highly differentiated sites from both HighD analyses and PCAdapt. The highlighted variants are the ones shared among IF2, IF3 and IF4.

POP-pair	SNP	CHR	Location	Ancestral_allele	Derived_allele	General_DAF	Isolate_DAF	Delta_DAF	HGNC symbol	Consequence	CADD score
IF1-ITG	rs112863601	2	208802168	C	T	0.311	0.717	0.405	PLEKHM3	intron_variant	2.454
IF1-ITG	rs9828592	3	33044339	T	C	0.491	0.875	0.384	GLB1	intron_variant	2.785
IF1-ITG	rs1398759	3	124888905	G	C	0.434	0.817	0.383	SLC12A8	intron_variant	1.764
IF1-ITG	rs1789693	11	74887165	A	T	0.250	0.708	0.458	SLCO2B1	intron_variant	7.651
IF1-ITG	rs28520541	12	121997478	A	G	0.208	0.575	0.368	KDM2B	intron_variant	6.89
IF1-ITG	rs2389240	13	96132701	A	C	0.250	0.625	0.375	CLDN10-AS1	non_coding_transcript_variant	3.996
IF1-ITG	rs3843738	17	43739194	A	G	0.316	0.683	0.367	RP11-105N13.4	non_coding_transcript_variant	8.144
IF1-ITG	rs55893840	17	71000371	A	G	0.255	0.717	0.462	SLC39A11	intron_variant	0.382
IF2-ITG	rs7415711	1	86457896	G	C	0.415	0.833	0.418	COL24A1	intron_variant	0.195
IF2-ITG	rs13391086	2	29615864	C	T	0.028	0.400	0.372	ALK	intron_variant	1.049
IF2-ITG	rs11924625	3	33070158	A	T	0.326	0.700	0.375	GLB1	intron_variant	0.024
IF2-ITG	rs7660497	4	58973339	C	T	0.203	0.589	0.386	SRIP1	downstream_gene_variant	4.403
IF2-ITG	rs3113813	4	137859741	G	C	0.156	0.478	0.322	RP11-138I17.1	non_coding_transcript_variant	0.44
IF2-ITG	rs190605097	9	39002471	G	T	0.401	0.878	0.477	-	intergenic_variant	0.218
IF2-ITG	rs12789966	11	99041999	A	G	0.288	0.711	0.423	CNTN5	intron_variant	1.274
IF2-ITG	rs10130552	14	71085815	C	T	0.170	0.611	0.441	CTD-2540L5.6	non_coding_transcript_variant	3.316
IF2-ITG	rs34956586	17	4430958	T	C	0.283	0.722	0.439	SPNS2	intron_variant	4.955
IF2-ITG	rs55893840	17	71000371	A	G	0.255	0.633	0.379	SLC39A11	intron_variant	0.382
IF3-ITG	rs13391086	2	29615864	C	T	0.028	0.436	0.408	ALK	intron_variant	1.049
IF3-ITG	rs1493927	3	19340911	C	T	0.316	0.745	0.429	KCNH8	intron_variant	1.357
IF3-ITG	rs6549575	3	67061960	G	A	0.349	0.766	0.417	KBTBD8	downstream_gene_variant	3.003
IF3-ITG	rs4947937	7	50907588	C	A	0.344	0.840	0.496	AC004920.3	non_coding_transcript_variant	2.682
IF3-ITG	rs35587464	8	121924092	C	T	0.274	0.670	0.397	RP11-369K17.1	upstream_gene_variant	3.961
IF3-ITG	rs7304148	12	10870231	T	C	0.226	0.617	0.391	YBX3	intron_variant	10.07
IF3-ITG	rs1525947	12	119456896	C	T	0.184	0.670	0.486	SRRM4	intron_variant	3.787
IF3-ITG	rs10130552	14	71085815	C	T	0.170	0.617	0.447	CTD-2540L5.6	non_coding_transcript_variant	3.316
IF3-ITG	rs1119141	16	84437223	T	C	0.406	0.798	0.392	ATP2C2	intron_variant	1.02
IF3-ITG	rs55893840	17	71000371	A	G	0.255	0.596	0.341	SLC39A11	intron_variant	0.382
IF3-ITG	rs67719508	20	33487278	T	C	0.212	0.660	0.447	ACSS2	intron_variant	1.417
IF3-ITG	rs1153336	21	41157658	C	G	0.203	0.638	0.436	IGSF5	intron_variant	1.322
IF3-ITG	rs2839327	21	47982652	A	G	0.175	0.553	0.379	DIP2A	intron_variant	0.634
IF4-ITG	rs13391086	2	29615864	C	T	0.028	0.431	0.402	ALK	intron_variant	1.049
IF4-ITG	rs6734194	2	153466691	G	T	0.387	0.847	0.460	FMNL2	intron_variant	3.689
IF4-ITG	rs3020453	3	39325523	T	C	0.113	0.597	0.484	CX3CR1	upstream_gene_variant	2.529
IF4-ITG	rs3113813	4	137859741	G	C	0.156	0.597	0.442	RP11-138I17.1	non_coding_transcript_variant	0.44
IF4-ITG	rs434602	6	6165468	T	C	0.307	0.736	0.430	F13A1	intron_variant	2.308
IF4-ITG	rs4475409	7	83621932	T	C	0.245	0.667	0.421	SEMA3A	intron_variant	0.982
IF4-ITG	rs2469386	8	3515312	C	A	0.231	0.708	0.477	CSMD1	intron_variant	0.515
IF4-ITG	rs7092649	10	60005202	G	A	0.198	0.639	0.441	IPMK	intron_variant	1.301
IF4-ITG	rs11222788	11	131649367	C	G	0.137	0.597	0.460	NTM	intron_variant	2.81
IF4-ITG	rs199984077	13	110078785	T	C	0.142	0.639	0.497	-	intergenic_variant	1.337
IF4-ITG	rs10130552	14	71085815	C	T	0.170	0.667	0.497	CTD-2540L5.6	non_coding_transcript_variant	3.316
IF4-ITG	rs8037845	15	93805290	C	G	0.156	0.569	0.414	RP11-326A13.1	downstream_gene_variant	0.105
IF4-ITG	rs191732434	16	3131937	A	C	0.288	0.792	0.504	RP11-473M20.9	non_coding_transcript_variant	0.132
IF4-ITG	rs4843293	16	88028003	G	A	0.363	0.750	0.387	BANP	intron_variant	1.159
IF4-ITG	rs34956586	17	4430958	T	C	0.283	0.611	0.328	SPNS2	intron_variant	4.955
IF4-ITG	rs55893840	17	71000371	A	G	0.255	0.625	0.370	SLC39A11	intron_variant	0.382
IF4-ITG	rs67719508	20	33487278	T	C	0.212	0.583	0.371	ACSS2	intron_variant	1.417
IF4-ITG	rs140038	22	36964359	C	T	0.340	0.778	0.438	CACNG2	intron_variant	6.845

PCAdapt-fast version was applied to each pair of populations separately (one isolate and its corresponding general population) using the whole-sample dataset for variants with MAF >0.05. Subsequently the p-values were transformed into q-values using the R package qvalue (http://github.com/jdstorey/qvalue) to filter the SNPs with false discovery rate (FDR) <0.1. All the variants were further filtered by requiring the derived allele frequency in isolates to be > 0.30. In total, 1077 sites met these criteria, with IF4 having the most; we did not find any sites in FIK, IVB and GRM (Supplementary Data). 39 of these sites overlapped with the HighD sites. We did not find any missense, LoF or other coding functional changes in the overlap, but three SNPs had

CADD scores greater than 5, indicating that they are potentially functionally important. The most interesting finding from these analyses was that six of these variants are shared between different isolates from Italy: IF2, IF3 and IF4. We interpret these as sites that were potentially positively selected in the ITG for the ancestral allele after the population split from the isolates. However, the underlying selection force is unclear. Four SNPs lie in the protein-coding genes *ALK*, *SPNS2*, *SLC39A11* and *ACSS2*, and may merit future follow-up. *ALK* is a gene involved in obesity[22] and glucose homeostasis[23] traits; *SPNS2* is also implicated in obesity[24]. *SLC39A11* was linked to pathways associated with relative hand skill[25], and finally *ACSS2* was linked to protein C levels[26].

Supplementary Notes

Variant calling and counts

SNP site selection

SNP sites were included based on the following cumulative strategy (i.e. a + b + c): a) all sites in the isolates: FIK, GRM, IF1, IF2, IF3, IF4, IVB and UKO and the general population FIG. b) all sites in the 1000 Genomes Phase 3 populations, thus also including the Toscani from Italy (ITG, labelled as TSI in 1000 Genomes publications). c) all sites with a non-reference allele count, AC ≥5 in the UKG.

Additionally, we required a non-reference allele count, AC ≥1, within the input set of individuals, a technicality due to some call sets having been made together with external data, thus avoiding sites which are not polymorphic in the samples used. Only the autosomes were considered.

Genotype likelihood calculation

Genotype likelihoods were calculated with samtools/bcftools (0.2.0-rc9) on the dataset above, plus the 21 other worldwide populations in the 1000 Genomes Phase 3 data[19]:

```
samtools mpileup -IE -C50 -d100000 -t DP,DP4 -l wgs.isolates.union.AC1.vcf.gz
bcftools call -mAC alleles -f GQ,GP -T wgs.isolates.union.AC1.alleles.gz
```

We dropped three samples from IVB (EGAN00001098982, XX129575 and XX021810) and two samples from UKO (EGAN00001098982 and EGAN00001010505) due to their high ratio of heterozygous to homozygous calls compared to all other samples. This can be a sign of contamination or different ancestry.

Genotype calling

Genotypes were called and phased using Beagle v4 (r1274)[27]. The input genotype likelihood VCFs were split into regions containing a minimum of 3000 sites with 500 buffer sites on either side of the region.

```
java $jvm_args -jar b4.r1274.jar
        phase-its=5
        nthreads=12
        gl=$region.in.vcf.gz
        out=$region.out
```

The overlapping output region VCFs were then ligated to per-chromosome VCF files using ‘bcftools concat –l’.

Annotation

Only the INFO/DP and FORMAT/GT from the original vcf files were kept, while the INFO/AC, INFO/AN, INFO/AF, INFO/NS were added with bcftools to annotate the complete dataset. INFO/AA (ancestral allele) was added with fill-aa using files from the 1000 Genome Phase 3 ancestral allele file.

INFO/GERP was added using bcftools annotate.
The ID column was filled with rsIDs from dbSNP141 using bcftools annotate.
Variant Effect predictor (VEP) annotation from Ensembl 76 was added with:

```
variant_effect_predictor.pl
   --assembly GRCh37
   --everything
   --allele_number
   --plugin Condel,/path/to/config/Condel/config/,b
   --plugin Blosum62
   --plugin LoF, human_ancestor_fa:/path/to/human_ancestor.fa.rz
   --format vcf
   --vcf
   --cache
   --dir /path/to/vep_cache
   --no_progress
   --quiet
   --offline
   --force_overwrite
   --no_stats
```

including the LOFTEE plugin (https://github.com/konradjk/loftee) for identifying LoF (loss-of-function) variation.

Files and availability

- {CHROM}.ISOLATES.mpileup.beagle.anno.20140815.vcf.gz: phased genotype calls in VCF format
- {CHROM}.ISOLATES.mpileup.beagle.anno.20140815.bcf: phased genotype calls in BCF format
- {CHROM}.ISOLATES.mpileup.beagle.anno.20140815.sites.vcf.gz: sites-only VCF files
- {CHROM}.ISOLATES.mpileup.beagle.anno.20140815.vcf.gz.stats: stats file generated by `bcftools stats`
- {CHROM}.ISOLATES-summary.pdf: default summary slides from `bcftools stats`
- README.20140815: this file
- ISOLATES.panel: lists all 9,375 samples and their cohort
- ISOLATES.cohorts: lists the cohorts
- 1000G_related_individuals.txt: lists related individuals in the Phase3 release.
- UK10K_exclusion_from_association.txt: lists samples excluded from certain downstream UK10K analyses due to relatedness, non-European-ness, etc.

All of these files are publicly available from the EGA (accession number: EGAD00001002014) under managed access following completion of a data access agreement.

Variant calling for GRG

100 samples from the HELIC TEENAGE (TEENs of Attica: Genes and Environment) cohort composed of young adults from Athens, Greece, were sequenced at 30X depth using the Illumina HiSeq X Ten platform. Variants were called on a per-sample basis using samtools 0.1.18 against the union of all 29,210,157 sites that were called as non-monomorphic in the whole dataset in the section 1.2. The calling omitted indels and sites where read depth exceeded 3,000 times the average read depth (100,000 reads). Individual VCFs were then merged using bcftools. Across called variants, mean read depth was 32.4X.

Validation

To assess the performance of the genotype calling from the low coverage data, we compared the genotypes against genotype chip data available for a subset of the cohorts. Chip data was available for 1,772 samples in the 1000 Genomes Phase3 cohort, 489 samples in the SISu and Kuusamo cohorts (FIG and FIK) and 2,402 samples in the UK10K cohort (UKG). Discordance rates for each cohort on chromosome 20 are shown in Supplementary Table 2.

Consortium funding support

UK10K: *ALSPAC:* This study makes use of data generated by the UK10K Consortium. The Wellcome Trust provided funding for UK10K (WT091310). Medical Research Council (S. Ring, MC_UU_12015/2, MR/J012165/1 to L. Paternoster, MC_UU_12013/1-9 to N. J. Timpson, G. D. Smith, D. Evans, T. Gaunt, H. Shihab). The UK Medical Research Council and the Wellcome Trust (Grant ref: 102215/2/13/2) and the University of Bristol provide core support for ALSPAC. We are extremely grateful to all the families who took part in this study, the midwives for their help in recruiting them, and the whole ALSPAC team, which includes interviewers, computer and laboratory technicians, clerical workers, research scientists, volunteers, managers, receptionists and nurses. The UK Medical Research Council and the Wellcome Trust (Grant ref: 102215/2/13/2) and the University of Bristol provide core support for ALSPAC. This publication is the work of the authors and they will serve as guarantors for the contents of this paper. GWAS data was generated by Sample Logistics and Genotyping Facilities at the Wellcome Trust Sanger Institute and LabCorp (Laboratory Corportation of America) using support from 23andMe.
TwinsUK: TwinsUK receives support from the National Institute for Health Research (NIHR) BioResource Clinical Research Facility and Biomedical Research Centre based at Guy's and St Thomas' NHS Foundation Trust and King's College London, Wellcome Trust Sanger Institute and National Eye Institute. The Wellcome Trust provided funding for UK10K (WT091310). EU grant EU FP7 (257082, HEALTH-F5-2011-282510). We are extremely grateful to all the participants who took part in this study, those who helped recruitment and the whole TUK team.
ORCADES: ORCADES was supported by the Chief Scientist Office of the Scottish Government (CZB/4/276, CZB/4/710), the Royal Society, the MRC Human Genetics Unit, Arthritis Research UK and the European Union framework program 6 EUROSPAN project (contract no. LSHG-CT-2006-018947). DNA extractions were performed at the Wellcome Trust Clinical Research Facility in Edinburgh. The University of Edinburgh is a charitable body, registered in Scotland, with registration number SC005336.
HELIC MANOLIS: This work was funded by the Wellcome Trust (WT098051) and the European Research Council (ERC-2011-StG 280559-SEPI). The MANOLIS study is dedicated to the memory of Manolis Giannakakis, 1978-2010. We thank the residents of the Mylopotamos villages for taking part. The HELIC study has been supported by many individuals who have contributed to sample collection (including Antonis Athanasiadis, Olina Balafouti, Christina Batzaki, Georgios Daskalakis, Eleni Emmanouil, Pounar Feritoglou, Chrisoula Giannakaki, Margarita Giannakopoulou, Kiki Kaldaridou, Anastasia Kaparou, Vasiliki Kariakli, Stella Koinaki, Dimitra Kokori, Maria Konidari, Hara Koundouraki, Dimitris Koutoukidis, Vasiliki Mamakou, Eirini Mamalaki, Eirini Mpamiaki, Nilden Selim, Nesse Souloglou, Maria Tsoukana, Dimitra Tzakou, Katerina Vosdogianni, Niovi Xenaki, Eleni Zengini), data entry (Thanos Antonos, Dimitra Papagrigoriou, Betty Spiliopoulou), sample logistics (Sarah Edkins, Emma Gray), genotyping (Suzannah Bumpstead, Robert Andrews, Hannah Blackburn, Doug Simpkin, Siobhan Whitehead), research administration (Anja Kolb-Kokocinski, Carol Smee, Danielle Walker) and informatics (Kathleen Stirrups, Martin Pollard, Josh Randall).
SISu Consortium: The Sequencing Initiative Suomi (SISu) project is an international collaboration between research groups aiming to build tools for genomic medicine (www.sisuproject.fi). These groups are generating whole genome and whole exome sequence

data from Finnish samples and provide data resources for the research community. Key groups of the project are from Universities of Eastern Finland, Oulu and Helsinki and The Institute for Health and Welfare, Finland, Lund University, The Wellcome Trust Sanger Institute, University of Oxford, The Broad Institute of Harvard and MIT, University of Michigan, Washington University in St. Louis, and University of California, Los Angeles (UCLA). The project is coordinated in the Institute for Molecular Medicine Finland at the University of Helsinki. AP and SR are supported by the Academy of Finland (grant no. 251704, 286500, 293404 to AP, and 251217, 285380 to SR), the Wellcome Trust (WT089061 and WT089062), Juselius Foundation, Finnish Foundation for Cardiovascular Research and Biocentrum Helsinki (to SR). HC was supported by the Doctoral Programme in Biomedicine (DPBM), University of Helsinki. PP was supported by the Nordic Information for Action eScience Center NIASC/NordForsk (grant no. 62721 to AP, PP & SR) and by IUT20-60 Omics for health: an integrated approach to understand and predict human disease.

Data Access Agreement

WTSI Data Access Agreement

WELLCOME TRUST SANGER INSTITUTE

DATA ACCESS AGREEMENT (August 2014 v7)

These terms and conditions govern access to the managed access datasets (details of which are set out in Appendix I) to which the User Institution has requested access. The User Institution agrees to be bound by these terms and conditions.

Definitions

Authorised Personnel: The individuals at the User Institution to whom WTSI grants access to the Data. This includes the User, the individuals listed on the User Institution's initial request for access to the Data and any other individuals for whom the User Institution subsequently requests access to the Data. Details of the initial Authorised Personnel are set out in Appendix I.

Data: The managed access datasets to which the User Institution has requested access.

Data Producers: WTSI and the collaborators listed in Appendix I responsible for the development, organisation, and oversight of the Data.

External Collaborator: A collaborator of the User, working for an institution other than the User Institution.

Project: The project for which the User Institution has requested access to the Data. A description of the Project is set out in Appendix II.

Publications: Includes, without limitation, articles published in print journals, electronic journals, reviews, books, posters and other written and verbal presentations of research.

Research Participant: An individual whose data form part of the Data.

Research Purposes: shall mean research that is seeking to advance the understanding of genetics and genomics, including the treatment of disorders, and work on statistical methods that may be applied to such research.

User: The principal investigator for the Project.

User Institution(s): The Institution that has requested access to the Data.

WTSI: Genome Research Limited, operating as the Wellcome Trust Sanger

Wellcome Trust Sanger Institute, Genome Research Limited (reg no. 2742969) is a charity registered in England with number 1021457

Institute	

Terms of the Agreement

1. The User Institution agrees to only use the Data for the purpose of the Project (described in Appendix II) and only for Research Purposes. The User Institution further agrees that it will only use the Data for Research Purposes which are within the limitations (if any) set out in Appendix I.

2. The User Institution agrees to preserve, at all times, the confidentiality of the Data. In particular, it undertakes not to use, or attempt to use the Data to compromise or otherwise infringe the confidentiality of information on Research Participants. Without prejudice to the generality of the foregoing, the User Institution agrees to use at least the measures set out in Appendix I to protect the Data.

3. The User Institution agrees to protect the confidentiality of Research Participants in any research papers or publications that they prepare by taking all reasonable care to limit the possibility of identification.

4. The User Institution agrees not to link or combine the Data to other information or archived data available in a way that could re-identify the Research Participants, even if access to that data has been formally granted to the User Institution or is freely available without restriction.

5. The User Institution agrees only to transfer or disclose the Data, in whole or part, or any material derived from the Data, to the Authorised Personnel. Should the User Institution wish to share the Data with an External Collaborator, the External Collaborator must complete a separate application for access to the Data.

6. The User Institution agrees that the Data Producers, and all other parties involved in the creation, funding or protection of the Data: a) make no warranty or representation, express or implied as to the accuracy, quality or comprehensiveness of the Data; b) exclude to the fullest extent permitted by law all liability for actions, claims, proceedings, demands, losses (including but not limited to loss of profit), costs, awards damages and payments made by the Recipient that may arise (whether directly or indirectly) in any way whatsoever from the Recipient's use of the Data or from the unavailability of, or break in access to, the Data for whatever reason and; c) bear no responsibility for the further analysis or interpretation of these Data.

7. The User Institution agrees to follow the Fort Lauderdale Guidelines (http://www.wellcome.ac.uk/stellent/groups/corporatesite/@policy_communications/documents/web_document/wtd003207.pdf) and the Toronto Statement

(http://www.nature.com/nature/journal/v461/n7261/full/461168a.html). This includes but is not limited to recognising the contribution of the Data Producers and including a proper acknowledgement in all reports or publications resulting from the use of the Data.

8. The User Institution agrees to follow the Publication Policy in Appendix III. This includes respecting the moratorium period for the Data Producers to publish the first peer-reviewed report describing and analysing the Data.

9. The User Institution agrees not to make intellectual property claims on the Data and not to use intellectual property protection in ways that would prevent or block access to, or use of, any element of the Data, or conclusion drawn directly from the Data.

10. The User Institution can elect to perform further research that would add intellectual and resource capital to the data and decide to obtain intellectual property rights on these downstream discoveries. In this case, the User Institution agrees to implement licensing policies that will not obstruct further research and to follow the U.S. National Institutes of Health Best Practices for the Licensing of Genomic Inventions (2005) (https://www.icgc.org/files/daco/NIH_BestPracticesLicensingGenomicInventions_2005_en.pdf) in conformity with the Organisation for Economic Co-operation and Development Guidelines for the Licensing of the Genetic Inventions (2006) (http://www.oecd.org/science/biotech/36198812.pdf).

11. WTSI is funded by the Wellcome Trust whose charitable objective is to improve health. If results arising from the User Institution's use of the Data could provide health solutions for the benefit of people in the developing world, the User Institution agrees to offer non-exclusive licenses to such results on a reasonable basis for use in low income and low-middle income countries (as defined by the World Bank) to any party that requests such a license solely for uses within these territories.

12. The User Institution agrees to destroy/discard the Data held, once it is no longer used for the Project, unless obliged to retain the data for archival purposes in conformity with audit or legal requirements.

13. The User Institution will notify WTSI within 30 days of any changes or departures of Authorised Personnel.

14. The User Institution will notify WTSI prior to any significant changes to the protocol for the Project.

15. The User Institution will notify WTSI as soon as it becomes aware of a breach of the terms or conditions of this agreement.

16. WTSI may terminate this agreement by written notice to the User Institution. If this agreement terminates for any reason, the User Institution will be required to destroy any Data held, including copies and backup copies. This clause does not prevent the User Institution from retaining the data for archival purpose in conformity with audit or legal requirements.

17. The User Institution accepts that it may be necessary for the Data Producers to alter the terms of this agreement from time to time. As an example, this may include specific provisions relating to the Data required by Data Producers other than WTSI. In the event that changes are required, the Data Producers or their appointed agent will contact the User Institution to inform it of the changes and the User Institution may elect to accept the changes or terminate the agreement.

18. If requested, the User Institution will allow data security and management documentation to be inspected to verify that it is complying with the terms of this agreement.

19. The User Institution agrees to distribute a copy of these terms to the Authorised Personnel. The User Institution will procure that the Authorised Personnel comply with the terms of this agreement.

20. This agreement (and any dispute, controversy, proceedings or claim of whatever nature arising out of this agreement or its formation) shall be construed, interpreted and governed by the laws of England and Wales and shall be subject to the exclusive jurisdiction of the English courts.

APPENDIX I - DATASET DETAILS

Dataset Reference(s)
FAKE_EGA_ID:2569827c-94f9-4bdc-8c7c-d946a30711c0

Name of project that created the dataset
Low-depth whole genome sequencing across multiple isolated populations

Names of other data producers

Daniela Toniolo	DIBIT-San Raffaele Scientific Institute Milano
Veikko Salomaa	National Institute for Health and Welfare, Finland (THL)
Dr. Satu Mannisto	National Institute for Health and Welfare, Finland (THL)
George Dedoussis	Harokopio University, Athens
Paolo Gasparini	Trieste University
Dr. Jim Wilson	The University of Edinburgh
Professor George Davey Smith	University of Bristol
Tim Spector	KCL

Aarno Palotie Institute for Molecular Medicine Finland (FIMM), The Broad Institute of MIT and Harvard

Specific limitations on areas of research

None.

Minimum protection measures required

Security Level: 2

File access: Data can be held in unencrypted files on an institutional compute system, with Unix user group read/write access for one or more appropriate groups but not Unix world read/write access behind a secure firewall. Laptops holding this data should have password protected logins and screenlocks (set to lock after 5 min of inactivity). If held on USB keys or other portable hard drives, the data must be encrypted.

APPENDIX II - PROJECT DETAILS

Details of dataset(s)
FAKE_EGA_ID:2569827c-94f9-4bdc-8c7c-d946a30711c0

Description of the project
-- TEST --

User Institution

Affiliation: Wellcome Trust Sanger Institute

Mailing Address: Wellcome Trust Sanger Institute, Genome Campus, Hinxton, CB101SA, United Kingdom

Principal Investigator: Stephen Rice

Individuals who the User Institution wishes to have access to the Data

APPENDIX III - PUBLICATION POLICY

WTSI are committed to the principles of rapid data release. WTSI intend to publish the results of our analysis of this data set and do not consider its deposition into public databases to be the equivalent of such publications. WTSI anticipate that the data set could be useful to other qualified researchers for a variety of purposes. However, some areas of work are therefore subject to a publication moratorium.

The publication moratorium covers any publications (including oral

communications) that describe the use of the dataset. For research papers, submission for publication should not occur until 12 months after these data were first made available on the relevant hosting database, unless WTSI has provided written consent to earlier submission.

In any publications based on this data, please describe how the data can be accessed, including the name of the hosting database (e.g., The European Genome-phenome Archive at the European Bioinformatics Institute) and its accession numbers (e.g., EGAS00000000029), and acknowledge its use in a form agreed by the User Institution with WTSI.

Supplementary References

1. Price, A.L. *et al.* Principal components analysis corrects for stratification in genome-wide association studies. *Nat Genet* **38**, 904-909 (2006).
2. Alexander, D.H., Novembre, J. & Lange, K. Fast model-based estimation of ancestry in unrelated individuals. *Genome Res* **19**, 1655-1664 (2009).
3. Pickrell, J.K. & Pritchard, J.K. Inference of population splits and mixtures from genome-wide allele frequency data. *PLoS Genet* **8**, e1002967 (2012).
4. Hill, W.G. Estimation of effective population size from data on linkage disequilibrium. *Genetical Res.* **38**, 209-216 (1981).
5. Hayes, B.J., Visscher, P.M., McPartlan, H.C. & Goddard, M.E. Novel multilocus measure of linkage disequilibrium to estimate past effective population size. *Genome Res* **13**, 635-643 (2003).
6. Tenesa, A. *et al.* Recent human effective population size estimated from linkage disequilibrium. *Genome Res* **17**, 520-526 (2007).
7. Mezzavilla, M. & Ghirotto, S. *Neon*: An R package to estimate human effective population size and divergence time from patterns of linkage disequilibrium between SNPs. *J Comput Sci Syst Biol* **8**, 37-44 (2015).
8. Schiffels, S. & Durbin, R. Inferring human population size and separation history from multiple genome sequences. *Nat Genet* **46**, 919-925 (2014).
9. Browning, S.R. & Browning, B.L. Accurate non-parametric estimation of recent effective population size from segments of identity by descent. *Am J Hum Genet* **97**, 404-418 (2015).
10. Browning, B.L. & Browning, S.R. Detecting identity by descent and estimating genotype error rates in sequence data. *Am J Hum Genet* **93**, 840-851 (2013).
11. Benazzo, A., Panziera, A. & Bertorelle, G. 4P: fast computing of population genetics statistics from large DNA polymorphism panels. *Ecol Evol* **5**, 172-175 (2014).
12. Schliep, K.P. phangorn: phylogenetic analysis in R. *Bioinformatics* **27**, 592-3 (2011).
13. Purcell, S. *et al.* PLINK: a tool set for whole-genome association and population-based linkage analyses. *Am J Hum Genet* **81**, 559-575 (2007).
14. Polasek, O. *et al.* Comparative assessment of methods for estimating individual genome-wide homozygosity-by-descent from human genomic data. *BMC Genomics* **11**, 139 (2010).
15. Gabriel, S.B. *et al.* The structure of haplotype blocks in the human genome. *Science* **296**, 2225-2229 (2002).
16. Wang, H. & Song, M. Ckmeans.1d.dp: optimal k-means clustering in one dimension by dynamic programming. *The R Journal* **3**, 29-33 (2011).
17. Colonna, V. *et al.* Human genomic regions with exceptionally high levels of population differentiation identified from 911 whole-genome sequences. *Genome Biol* **15**, R88 (2014).
18. Field, Y. *et al.* Detection of human adaptation during the past 2000 years. *Science* **354**, 760-764 (2016).
19. The 1000 Genomes Project Consortium. A global reference for human genetic variation. *Nature* **526**, 68-74 (2015).

20. The UK10K Consortium. The UK10K project identifies rare variants in health and disease. *Nature* **526**, 82-90 (2015).
21. Do, R. *et al.* No evidence that selection has been less effective at removing deleterious mutations in Europeans than in Africans. *Nat Genet* **47**, 126-131 (2015).
22. Locke, A.E. *et al.* Genetic studies of body mass index yield new insights for obesity biology. *Nature* **518**, 197-206 (2015).
23. Palmer, N.D. *et al.* Genetic variants associated with quantitative glucose homeostasis traits translate to Type 2 Diabetes in Mexican Americans: The GUARDIAN (Genetics Underlying Diabetes in Hispanics) Consortium. *Diabetes* **64**, 1853-1866 (2015).
24. Comuzzie, A.G. *et al.* Novel genetic loci identified for the pathophysiology of childhood obesity in the Hispanic population. *PLoS One* **7**, e51954 (2012).
25. Brandler, W.M. *et al.* Common variants in left/right asymmetry genes and pathways are associated with relative hand skill. *PLoS Genet* **9**, e1003751 (2013).
26. Athanasiadis, G. *et al.* A genome-wide association study of the Protein C anticoagulant pathway. *PLoS One* **6**, e29168 (2011).
27. Browning, S.R. & Browning, B.L. Rapid and accurate haplotype phasing and missing-data inference for whole-genome association studies by use of localized haplotype clustering. *Am J Hum Genet* **81**, 1084-1097 (2007).
